# CircNTNG1 inhibits renal cell carcinoma progression via HOXA5-mediated epigenetic silencing of Slug

**DOI:** 10.1186/s12943-022-01694-7

**Published:** 2022-12-19

**Authors:** Yanping Liang, Junjie Cen, Yong Huang, Yong Fang, Yunfei Wang, Guannan Shu, Yihui Pan, Kangbo Huang, Jiaqi Dong, Mi Zhou, Yi Xu, Junhang Luo, Min Liu, Jiaxing Zhang

**Affiliations:** 1 Department of Laboratory Medicine, The First Affiliated Hospital, Sun Yat-sen University, Guangzhou, 510080 China; 2 Department of Urology, The First Affiliated Hospital, Sun Yat-sen University, Guangzhou, 510080 China; 3 Department of Emergency, The First Affiliated Hospital, Sun Yat-sen University, Guangzhou, 510080 China; 4 Department of Oncology, The First Affiliated Hospital, Sun Yat-sen University, Guangzhou, 510080 China; 5 Department of Urology, Sun Yat-sen University Cancer Center, Guangzhou, 510060 China; 6 Institute of Precision Medicine, The First Affiliated Hospital, Sun Yat-sen University, Guangzhou, 510080 China

**Keywords:** Renal cell carcinoma, circNTNG1, miR-19b-3p, HOXA5, EMT pathway

## Abstract

**Background:**

Recent studies have identified that circular RNAs (circRNAs) have an important role in cancer via their well-recognized sponge effect on miRNAs, which regulates a large variety of cancer-related genes. However, only a few circRNAs have been well-studied in renal cell carcinoma (RCC) and their regulatory function remains largely elusive.

**Methods:**

Bioinformatics approaches were used to characterize the differentially expressed circRNAs in our own circRNA-sequencing dataset, as well as two public circRNA microarray datasets. CircNTNG1 (hsa_circ_0002286) was identified as a potential tumor-suppressing circRNA. Transwell assay and CCK-8 assay were used to assess phenotypic changes. RNA pull-down, luciferase reporter assays and FISH experiment were used to confirm the interactions among circNTNG1, miR-19b-3p, and HOXA5 mRNA. GSEA was performed to explore the downstream pathway regulated by HOXA5. Immunoblotting, chromatin immunoprecipitation, and methylated DNA immunoprecipitation were used to study the mechanism of HOXA5.

**Results:**

In all three circRNA datasets, circNTNG1, which was frequently deleted in RCC, showed significantly low expression in the tumor group. The basic properties of circNTNG1 were characterized, and phenotype studies also demonstrated the inhibitory effect of circNTNG1 on RCC cell aggressiveness. Clinically, circNTNG1 expression was associated with RCC stage and Fuhrman grade, and it also served as an independent predictive factor for both OS and RFS of RCC patients. Next, the sponge effect of circNTNG1 on miR-19b-3p and the inhibition of HOXA5 by miR-19b-3p were validated. GSEA analysis indicated that HOXA5 could inactivate the epithelial–mesenchymal transition (EMT) process, and this inactivation was mediated by HOXA5-induced SNAI2 (Slug) downregulation. Finally, it was confirmed that the Slug downregulation was caused by HOXA5, along with the DNA methyltransferase DNMT3A, binding to its promoter region and increasing the methylation level.

**Conclusions:**

Based on the experimental data, in RCC, circNTNG1/miR-19b-3p/HOXA5 axis can regulate the epigenetic silencing of Slug, thus interfering EMT and metastasis of RCC. Together, our findings provide potential biomarkers and novel therapeutic targets for future study in RCC.

**Supplementary Information:**

The online version contains supplementary material available at 10.1186/s12943-022-01694-7.

## Background

Renal cell carcinoma (RCC) is one of the ten most common adult malignancies, and its incidence has been increasing over the past 20 years [1]. RCC has a variety of histological subtypes. Clear cell RCC (ccRCC) is the most common subtype, consisting of nearly 80% of all RCC cases [2]. The main therapy for primary RCC is radical nephrectomy. However, nearly one third of patients will eventually relapse despite prompt surgery [3]. Thus, identifying key mechanisms that regulate RCC progression is essential for recurrence prediction and potential therapy of RCC.

In mammalian cells, gene expression is regulated by a complex network. A major component of this network is non-coding RNA (ncRNA). Approximately 80% of the human genome sequence can be transcribed into ncRNA [4]. Circular RNA (circRNA) is an important ncRNA subfamily, and it has a unique covalent, single-stranded, closed-loop structure, which lacks the canonical 5′ cap and 3′ poly-A tail of messenger RNA (mRNA) [5]. High-throughput sequencing has identified that many circRNAs have key physiological and pathological roles in cells [5]. During RCC development and progression, certain circRNAs have indispensable roles. For example, the circRNA cRAPGEF5 suppresses both RCC proliferation and metastasis by regulating the miR-27a-3p/TXNIP pathway [6]. CircSDHC promotes RCC progression via its sponge effect on miR-127-3p [7]. However, the function of circRNA in RCC remains largely elusive.

The homeobox (HOX) gene family consists of several gene subsets, including HOXA, HOXB, HOXC, and HOXD. These genes mainly encode transcription factors, which regulate cell proliferation and differentiation during embryogenesis [8]. One member of the family, HOXA5, is located on the A cluster of chromosome 7 and contains a conserved DNA-binding domain [9, 10]. HOXA5 plays an important role during respiratory system embryogenesis [11]. It is also involved in the regulation of various cancers. For example, in normal intestinal epithelial cells, HOXA5 is a potent inhibitor of Wnt signaling, keeping the cells differentiated. Once the function of HOXA5 is lost and Wnt signaling is disinhibited, the cells acquire a stem-like pattern and eventually develop into colorectal cancer [10]. However, in acute myeloid leukemia, HOXA5 upregulation is associated with poor outcomes [8]. These findings show that HOXA5 has complicated, even contradictory, functions in different cancers. The role of HOXA5 in RCC is currently largely unknown.

In this study, by analyzing three RCC circRNA datasets, we discovered the tumor-suppressing circRNA circNTNG1. The inhibitory role of circNTNG1 in RCC was investigated, both phenotypically and mechanistically. CircNTNG1/miR-19b-3p/HOXA5 axis was studied and found to regulate the epigenetic silencing of Slug, thus interfering EMT and progression of RCC. Together, our study provides information on potential biomarkers and novel therapeutic targets in RCC.

## Methods

### Clinical samples and data

Clinical and genomic data of The Cancer Genome Atlas (TCGA) kidney renal clear cell carcinoma (KIRC, i.e., ccRCC), kidney renal papillary cell carcinoma (KIRP), and kidney chromophobe (KICH) datasets were obtained from cBioPortal (https://www.cbioportal.org/). The chromatin immunoprecipitation sequencing (ChIP-seq) dataset GSE170384 was obtained from the ENCODE Project website (https://www.encodeproject.org/) and used as a reference to analyze the HOXA5-binding loci [12]. The circRNA microarray datasets GSE137836 (three matched primary and metastatic renal cancers) and GSE100186 (four matched primary renal cancers and adjacent tissues) were obtained from Gene Expression Omnibus (GEO; https://www.ncbi.nlm.nih.gov/geo/).

Regarding our own patient cohort, matched tumors and normal adjacent normal tissues from 102 ccRCC patients who had undergone surgery from December 2007 to December 2018 were collected from Sun Yat-sen University Cancer Center (Guangzhou, China), along with clinical data. Five pairs of matched tumor and adjacent normal tissue of ccRCC patients from the First Affiliated Hospital of Sun Yat-sen University (Guangzhou, China) were collected and subjected to circRNA-sequencing. The detailed clinical information of these 5 patients was listed in Additional file 7: Table S2. A metastatic RCC cohort, which contains renal tumor tissues from 14 RCC patients with distant metastasis at the time of diagnosis and 14 matched RCC patients without distant metastasis, were collected from the above two hospitals. Overall survival (OS) was defined as the time period from the date of surgery to the date of patient’s death for any reason. Recurrent-free survival (RFS) was defined as the time period from the date of surgery to the date of first clinical notice of recurrence. Informed consents were obtained from the patients. The usage of the samples and clinical data were approved by the Ethical Committees of Sun Yat-sen University Cancer Center and First Affiliated Hospital of Sun Yat-sen University.

### Bioinformatics analysis

Mutational status of genes in five ccRCC datasets (DFCI, BGI, IRC, TCGA, and UTokyo) and methylation level of genes in the TCGA KIRC dataset were analyzed using the cBioPortal platform (https://www.cbioportal.org/). GEPIA2 database (http://gepia2.cancer-pku.cn/#index) was used to conduct a survival profile analysis of HOXA family members, single-gene survival analyses, and gene expression correlation analyses [13]. The JASPAR transcription factor database (http://jaspar.genereg.net/) was queried to obtain the DNA-binding sequence of HOXA5. Gene Ontology (GO) enrichment analysis was conducted using the DAVID computational workflow (https://david.ncifcrf.gov/). CpG island prediction and primer design were performed using the MethPrimer website (https://www.urogene.org/methprimer/) [14]. R v4.0.2 (https://www.r-project.org/) was used to analyze differential gene expression (with limma package), construct GO analysis bubble plots (with ggplot2 package), and construct a circRNA-miRNA-mRNA network (with visNetwork package). Gene set enrichment analysis (GSEA; https://www.gsea-msigdb.org/gsea/index.jsp) was utilized to screen for the differential pathways between the high/low HOXA5 expression patient groups.

To identify frequently deleted circRNAs, differential analysis of expression was first performed. Thereafter, the circRNAs with circBase annotation that were also located in frequently deleted chromosome loci (1, 3p, 8, 9, and 22) [15–17] were selected. To predict the potential circRNA/miRNA interactions, the overlapping predictions of four algorithms (SVMicrO [18], DIANA-microT [19], ENCORI [20], and miRanda [21]) were selected, yielding the interactions with the greatest likelihood of being accurate. Similarly, to predict significant miRNA/mRNA interactions, the same four algorithms were used, and the overlapping results were chosen. A circNTNG1/miRNA/mRNA interaction network was established based on these significant circNTNG1/miRNA/mRNA interactions. Survival information for the predicted miRNAs was obtained from the ENCORI website (https://starbase.sysu.edu.cn/). The miRNA-regulating pathways were predicted using the mirPath v3 algorithm [22].

### Chromatin immunoprecipitation (ChIP) assay

ChIP assays were performed using a SimpleChIP Enzymatic Chromatin IP Kit (Cell Signaling Technology, MA, USA). Cells were crosslinked with 4% polyformaldehyde (Biosharp, Anhui, China) for 10 min, treated with glycine, washed with cold PBS, lysed, and subjected to micrococcal nuclease to digest the DNA to 150–900 bp fragments. The fragments were immunoprecipitated overnight at 4 °C with antibodies against specific protein or IgG control. The ChIP-enriched DNAs underwent qRT-PCR (primers are listed in Additional file 6: Table S1). Data was normalized to input. Antibodies used in ChIP are as followed: Flag (F7425, Sigma-Aldrich, MO, USA), DNMT3A (20954–1-AP, Proteintech, Wuhan, China).

### Methylated DNA immunoprecipitation (MeDIP) assay

MeDIP assays were performed with an EpiQuik MeDIP Ultra Kit (Epigentek, NY, USA) according to the manufacturer’s protocol. First, cells were lysed and sonicated to obtain fragmented DNA. The lysates were then added to anti-5-methylcytosine antibody- or control IgG antibody-coated wells. After hybridization, the DNA from the complexes was released, collected, eluted, and subjected to qRT-PCR (primers are listed in Additional file 6: Table S1) to determine the methylation level.

### RNA pull-down (RIP) assay

RNA pull-down assays were performed as previously described [23]. A specific DNA probe and a scrambled probe (control) were used. Briefly, the probes were biotinylated before co-incubating with total RNA extracts. The hybridized complex was then pulled down using streptavidin magnetic beads (Invitrogen, CA, USA). After purification, the RNA products were subjected to qRT-PCR.

### Animal experiment

The animal experiments were approved by the Institutional Animal Care and Use Committee of Sun Yat-sen University. A mouse tail vein injection model was used to study the in vivo phenotype of RCC cells. Briefly, nude BALB/c mice (3 weeks old; GemPharmatech, Guangdong, China) were randomly divided into experiment and control groups (8 mice/group). Cells were co-transfected with a luciferase-expressing plasmid and then injected into the mice circulation via the tail vein (2 × 10^6^ cells per mouse), allowing them to metastasize to the lungs. The in vivo luciferase activities were measured and representative photos were captured weekly using an IVIS Spectrum In Vivo Imaging System (PerkinElmer, MA, USA). After 8 weeks, the mice were euthanized and the lungs were subjected to HE and immunohistochemistry (IHC) staining. Sections were examined for pulmonary metastatic foci and representative photos were captured with an IX83 inverted microscope (Olympus, Shibuya-ku, Japan).

### Statistical analysis

Statistical analyses were conducted and graphs were constructed using GraphPad Prism 7. The bioinformatics analyses utilized R 4.0.2 (https://www.rproject.org/). Pairs of groups were compared by Student’s t-test. Correlation between continuous variables was calculated by Pearson’s correlation analysis. Correlation between categorial variables was test with chi-square test (with or without Yates’ continuity correction). Survival analysis was conducted using Kaplan–Meier curves and the logrank test. Univariate and multivariate analysis were conducted with Cox regression. When defining high or low expression groups, median expressions of circNTNG1, miR-19b-3p and HOXA5 were used as cut-off points. All experiments were conducted at least three times. Quantitative data are presented as mean ± SD. *P* < 0.05 was considered significant (*P* < 0.05 for *; *P* < 0.01 for **, *P* < 0.001 for ***).

## Results

### Sequencing and microarray datasets analysis reveals tumor-suppressing circNTNG1 in RCC

**Fig. 1:**
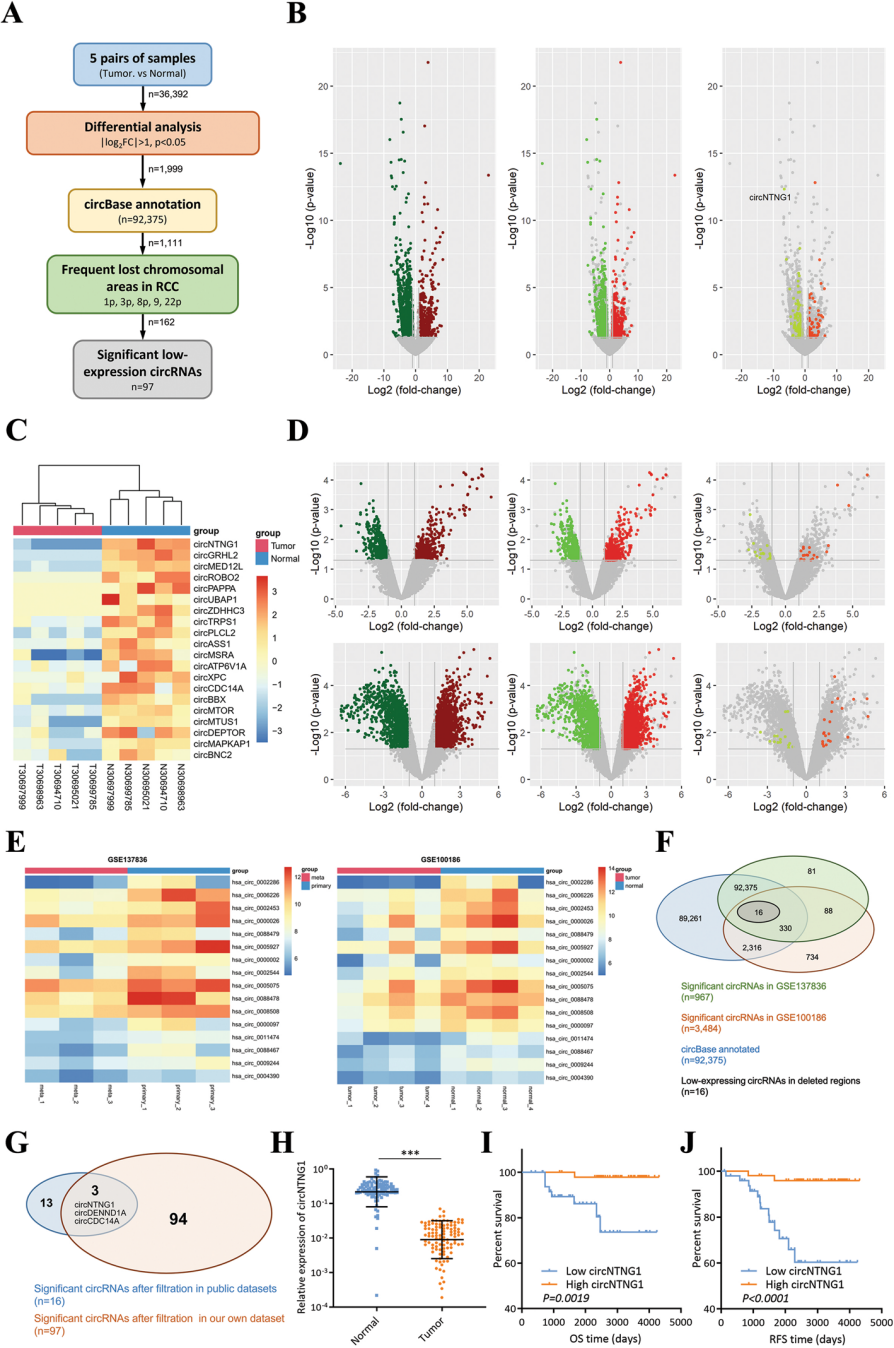
Sequencing and microarray datasets reveal tumor-suppressing circNTNG1 in RCC. **a**. Workflow of filtering frequently deleted circRNAs in RCC using our own patient dataset. Numbers near the arrows in each steps indicated the remaining circRNAs. **b**. Volcano plots of the remaining circRNAs during each step. Our own dataset was used. Colored dots indicated significant circRNAs. Grey dots indicated circRNAs that were filtered out. **c**. Heatmap of the top 20 frequently deleted circRNAs in our own circRNA sequencing dataset. **d**. Volcano plots of the remaining circRNAs during each step. Two public RCC circRNA microarray datasets were used. Colored dots indicated significant circRNAs. Grey dots indicated circRNAs that were filtered out. **e**. Heatmaps of the 16 frequently deleted circRNAs in two public RCC circRNA microarray datasets. **f**. Venn gram of the frequent deleted circRNAs selection in two public RCC circRNA microarray datasets. **g**. Venn gram of intersection of the two public RCC circRNA datasets and our own circRNA sequencing dataset. **h**. Expression analysis of circNTNG1 in tumors and normal adjacent tissues in our patient cohort (total 102 patients). **i**. Survival analysis (overall survival) of circNTNG1 in our patient cohort. **j**. Survival analysis (recurrent-free survival) of circNTNG1 in our patient cohort. Data are mean ± SD, *n* = 3

To explore meaningful circRNAs in RCC, five pairs of matched tumors and adjacent normal tissues of RCC patients collected from our hospital were subjected to circRNA sequencing (Additional file 7: Table S2). After selection of the circRNAs with significantly differential expression (*p* < 0.05, more than 2-fold change) and circBase annotation, 1111 circRNAs remained. Since more circRNAs appeared to be down-regulated in our dataset (643 down-regulated circRNAs vs. 468 up-regulated circRNAs), we decided to further focus on circRNAs with significant tumor-suppressing potentials. Thus, the circRNAs located on the frequently lost chromosome areas during RCC tumorigenesis (chromosome 1, 3p, 8, 9 and 22, Additional file 8: Table S3) were selected, yielding a final result of 97 circRNAs (Fig. 1A, Additional file 9: Table S4). For each step, the selected circRNAs were presented in volcano plots (Fig. 1B), and the top 20 significant down-regulated circRNAs were shown in a heatmap (Fig. 1C).

For external validation, two GEO circRNA microarray datasets on RCC (GSE137836 and GSE100186) were retrieved. After a similar analytic workflow (Additional file 1: Fig. S1A), 16 circRNAs that were located on frequently lost chromosomes and had significantly low expression in both datasets were identified (Additional file 10: Table S5). They were also showed in volcano plots and in a heatmap (Fig. 1D-F). Overlapping the 16 circRNAs with the 97 previously identified ones resulted in 3 circRNAs, among which the circNTNG1 was the top down-regulated circRNA in all three datasets, demonstrating > 90-fold and > 8-fold decrease in our sequencing dataset and two public datasets respectively (Fig. 1G).

In our RCC patient cohort, circNTNG1 was significantly down-regulated in tumor samples and lower expression of circNTNG1 was associated with worse OS and RFS (Fig. 1H-J). Furthermore, patients with more advanced clinical stages and higher Fuhrman grades had lower expression of circNTNG1 (Additional file 1: Fig. S1B, C). Also, RCC patients with distant metastasis demonstrated lower level of circNTNG1 in their renal tumors, compared to those without metastasis (Additional file 1: Fig. S1D). Chi-square analysis also discovered that circNTNG1 was significantly correlated with clinical stage and Fuhrman grade (Table 1). Finally, univariate and multivariate Cox analysis indicated that circNTNG1 was an independent prognostic factor for both OS and RFS (OS univariate HR: 0.1586, 95% CI: 0.03589–0.7005; OS multivariate HR: 0.2093, 95% CI: 0.04536–0.9657; RFS univariate HR: 0.1821, 95% CI: 0.06296–0.5267; RFS multivariate HR: 0.2454, 95% CI: 0.07676–0.7846) (Table 2 and Table 3). Collectively, these results showed that circNTNG1 primarily has a tumor-suppressing role in RCC.

**Table 1:** Association of circNTNG1 expression with clinicopathological characteristics in 102 ccRCC patients

Parameter	Total	circNTNG1 expression		***p*** value
		High	Low	
**Age(y)**				
< 60	71	40	31	0.08504
≥ 60	31	11	20	
**Gender**				
Female	34	21	13	0.5801
Male	68	30	38	
**Clinical (TNM) stage**				
I	66	45	21	1.884E-06^*^
II-III	36	6	30	
**Fuhrman grade**				
1 + 2	84	48	36	0.004276^*^
3 + 4	18	3	15	

* Pearson’s Chi-squared test with Yates’ continuity correction

**Table 2:** Univariate and multivariate Cox regression analyses of dfifferent parameters on overall survival

Parameter	Univariate Analysis		Multivariate Analysis	
	HR (95%CI)	***P*** Value	HR (95%CI)	***P*** Value
Age	0.9899 (0.9394–1.043)	0.7028	–	–
Gender (Female vs. male)	1.969 (0.4090–9.483)	0.3980	–	–
Clinical (TNM) stage (II-III vs. I)	2.044 (0.8058–5.187)	0.1322	–	–
Fuhrman (3 + 4 vs. 1 + 2)	4.2736 (1.676–10.89)	0.002350	2.845 (1.090–7.426)	0.03269
circNTNG1 expression (High vs. low)	0.1586 (0.03589–0.7005)	0.01511	0.2093 (0.04536–0.9657)	0.04499
HR = hazard ratio. CI = confidence interval

**Table 3:** Univariate and multivariate Cox regression analyses of dfifferent parameters on recurrent-free survival

Parameter	Univariate Analysis		Multivariate Analysis	
	HR (95%CI)	***P*** Value	HR (95%CI)	***P*** Value
Age	1.021 (0.9794–1.064)	0.3283	–	–
Gender (Female vs. male)	1.563 (0.4973–4.911)	0.4448	–	–
Clinical (TNM) stage (II-III vs. I)	2.986 (1.395–6.392)	0.004843	1.550 (0.6715–3.579)	0.3044
Fuhrman (3 + 4 vs. 1 + 2)	2.415 (1.122–5.195)	0.02412	1.335 (0.6032–2.955)	0.4759
circNTNG1 expression (High vs. low)	0.1821 (0.06296–0.5267)	0.001671	0.2454 (0.07676–0.7846)	0.01784
HR = hazard ratio. CI = confidence interval				

### Characteristics of circNTNG1 in RCC

**Fig. 2:**
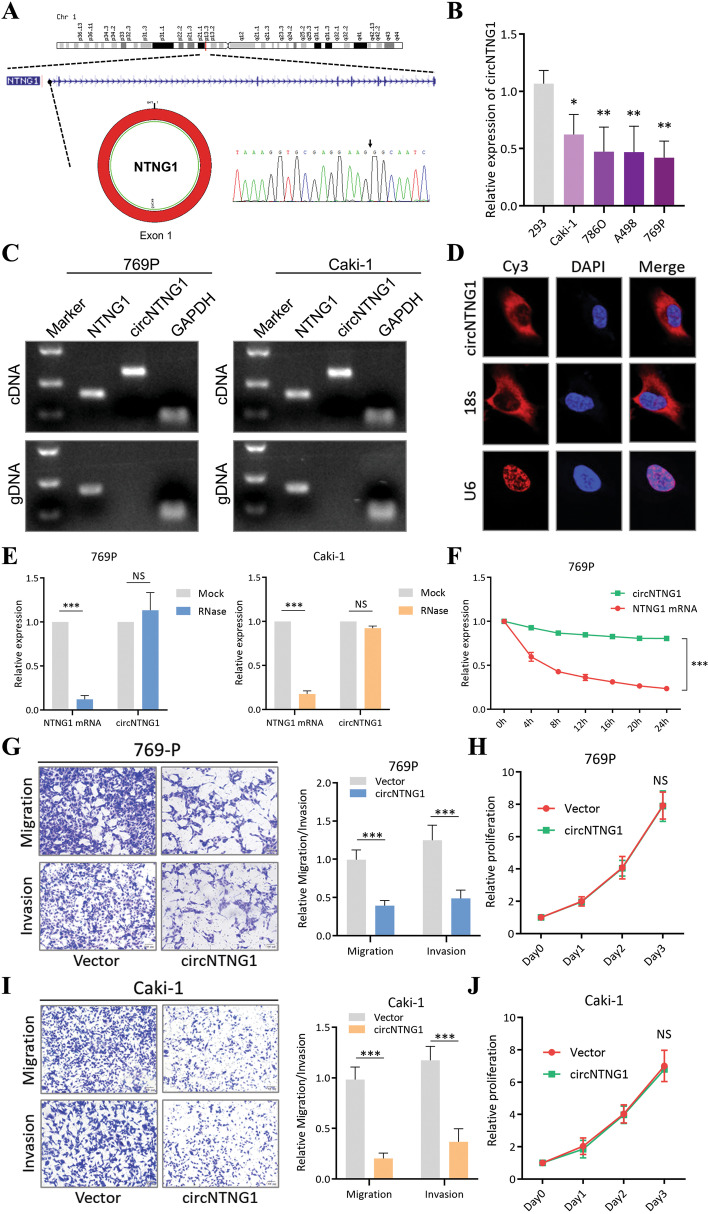
Characteristics of circNTNG1 in RCC. **a**. Chromosomal origin and Sanger sequencing confirmation of circNTNG1. **b**. Expression of circNTNG1 in normal kidney cell line 293 and four RCC cell lines (Caki-1, 786-O, A498 and 769P). **c**. DNA electrophoresis of circNTNG1 and linear NTNG1 from cDNA and gDNA in 769P and Caki-1. GAPDH was used as positive control. **d**. FISH experiment detecting the subcellular localization of circNTNG1. Eighteen s was used as positive cytoplasm control. U6 was used as positive nucleus control. **e**. RNase treatment assay of circNTNG1 and linear NTNG1 in 769P and Caki-1 cells. The RNA levels were determined by qRT-PCR. Expression levels were normalized to the mock group. **f**. Actinomycin D assays of circNTNG1 and linear NTNG1 in 769P cells. The RNA levels were determined by qRT-PCR. Expression levels were normalized to 0 h. **g**. Representative images (left) and quantification (right) data of Transwell migration/invasion assay of 769P cells with vector/circNTNG1-overexpression. Cell number was determined by counting five random fields under microscope. **h**. Proliferative activity of 769P cells with vector/circNTNG1-overexpression measured by CCK8 assay. Levels were normalized to day 0. **i**. Representative images (left) and quantification (right) data of Transwell migration/invasion assay of Caki-1 cells with vector/circNTNG1-overexpression. Cell number was determined by counting five random fields under microscope. **j**. Proliferative activity of Caki-1 cells with vector/circNTNG1-overexpression measured by CCK8 assay

CircNTNG1 (length: 641 bp; circBase ID: hsa_circ_0002286) is transcribed from the NTNG1 gene on chromosome 1, resulting from exon 1 back-splicing. Sanger sequencing confirmed the existence of this circRNA in RCC cells by identifying the back-splicing junction (Fig. 2A). Compared to normal kidney cell lines 293, four RCC cell lines (Caki-1, 786-O, A498 and 769P) showed circNTNG1 downregulation (Fig. 2B). Using a divergent primer, circNTNG1 could be amplified from cDNA but not gDNA, indicating that circNTNG1 was a result of splicing (Fig. 2C). Subcellular localization using FISH indicated that the majority of circNTNG1 localized to the cytoplasm (Fig. 2D). In contrast to the linear NTNG1 mRNA, circNTNG1 was more resistant to RNase and had a longer half-life (Fig. 2E, F). Overexpression of circNTNG1 decreased both migration and invasion abilities of 769P, Caki-1 and 786-O cells (Fig. 2G, I and Additional file 1: Fig. S1E). However, the proliferation ability was unchanged (Fig. 2H, J and Additional file 1: Fig. S1F).

### CircNTNG1 exerts a sponge effect on miR-19b-3p

**Fig. 3:**
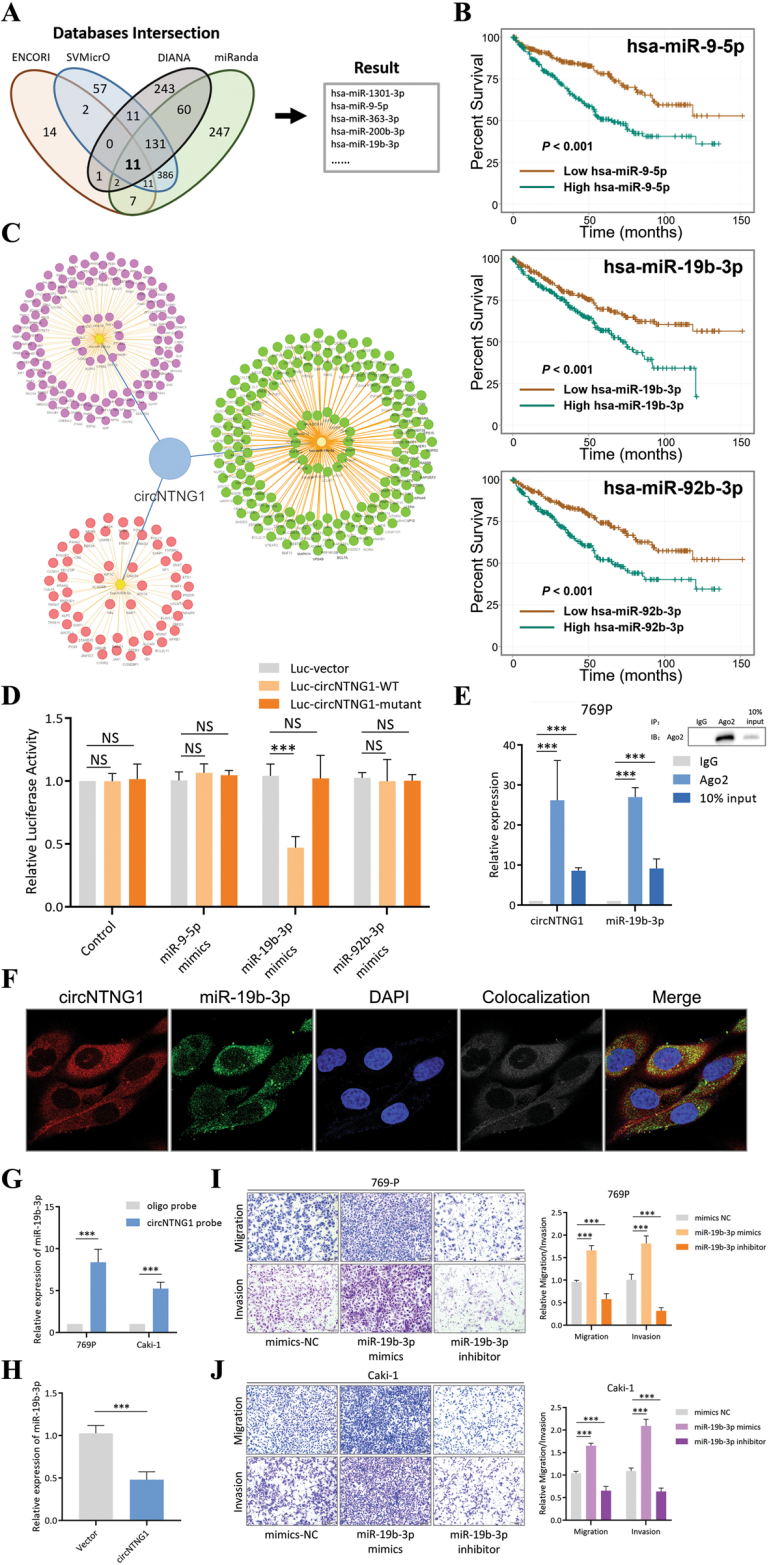
CircNTNG1 exerted a sponge effect on miR-19b-3p. **a**. Venn gram of finding the potential interacting miRNAs of a circNTNG1. The final miRNAs were generated by intersecting the results of four different online databases (SVMicrO, DIANA-microT, ENCORI, and miRanda). **b**. Survival analysis of three potential miRNAs involved in the circNTNG1 regulatory axis on TCGA KIRC patients. **c**. The circNTNG1/miRNA/mRNA interaction network. **d**. Luciferase reporter assay with vector, circNTNG1 wild-type sequence and circNTNG1 mutant sequence transfected with three different miRNA mimics. Vector group was used as normalization control. **e**. RNA immunoprecipitation using an Ago2 antibody and qRT-PCR quantification of circNTNG1 and miR-19b-3p in the pull-down product. IgG group was used as normalization control. **f**. FISH co-localization experiment of circNTNG1 and miR-19b-3p. DAPI was used to indicate the location of nuclei. **g**. RNA pull-down experiment with a circNTNG1 specific probe. An oligo probe was used a negative control. **h**. qRT-PCR quantification of miR-19b-3p in 769P cells transfected with control vector/circNTNG1 overexpression. The control vector group was used as normalization. **i**. Representative images (left) and quantification (right) data of Transwell migration/invasion assay of 769P cells with miR-19b-3p mimics/miR-19b-3p inhibitor. **j**. Representative images (left) and quantification (right) data of Transwell migration/invasion assay of Caki-1 cells with miR-19b-3p mimics/miR-19b-3p inhibitor. Data are mean ± SD, *n* = 3

One of the key functions of circRNAs is their ability to sponge specific miRNAs, which regulates a large variety of cancer-related genes [24]. We wondered whether circNTNG1 could also exhibit this sponge effect. To predict the potential circNTNG1/miRNA interactions, the overlapping predictions of four algorithms (SVMicrO, DIANA-microT, ENCORI and miRanda) were selected, yielding the interactions with the greatest likelihood of being accurate (Fig. 3A). Eleven miRNAs were found to be potentially interacting with circNTNG1, among which only hsa-miR-9-5p, hsa-miR-19b-3p and hsa-miR-92b-3p were significantly associated with poor survival in TCGA KIRC (ccRCC) dataset (Fig. 3B). The genes potentially regulated by these three miRNAs were also predicted with the same method, and a circNTNG1/miRNA/mRNA interaction network was constructed (Fig. 3C). Next, to further assess the circNTNG1-miRNA interactions, luciferase reporter plasmids containing critical wild-type and mutant sequences were constructed (Additional file 6: Table S1). It was validated that only miR-19b-3p could interact with circNTNG1 (Fig. 3D). Using an anti-Ago2 antibody in RIP assays confirmed that both circNTNG1 and miR-19b-3p could bind to Ago2 (Fig. 3E), which is essential for the circRNA/miRNA sponge effect [25]. Fluorescence in situ hybridization (FISH) assays showed that circNTNG1 and miR-19b-3p co-localized in the cytoplasm (Fig. 3F). Using a specific circNTNG1 probe compared to a control probe, a significant amount of miR-19b-3p was pulled down in both 769P and Caki-1 cells (Fig. 3G). Overexpression of circNTNG1 caused a significant down-regulation of miR-19b-3p (Fig. 3H). As predicted by mirPath, the major regulatory functions of miR-19b-3p were ion binding and nucleic acid binding (Additional file 2: Fig. S2A). In our patient cohort, miR-19b-3p demonstrated higher expression in tumor tissue, and was also significantly associated poor OS and RFS (Additional file 2: Fig. S2B-D). Also, the level of miR-19b-3p was higher in renal tumors of patients with distant metastasis (Additional file 2: Fig. S1E). Consistent with these findings, after applying a mimic or an inhibitor of miR-19b-3p, the migration and invasion changes of RCC cells showed corresponding changes, consistent with the oncogenic ability of miR-19b-3p (Fig. 3I, J).

### MiR-19b-3p regulates the progression of RCC through inhibiting expression of HOXA5

**Fig. 4:**
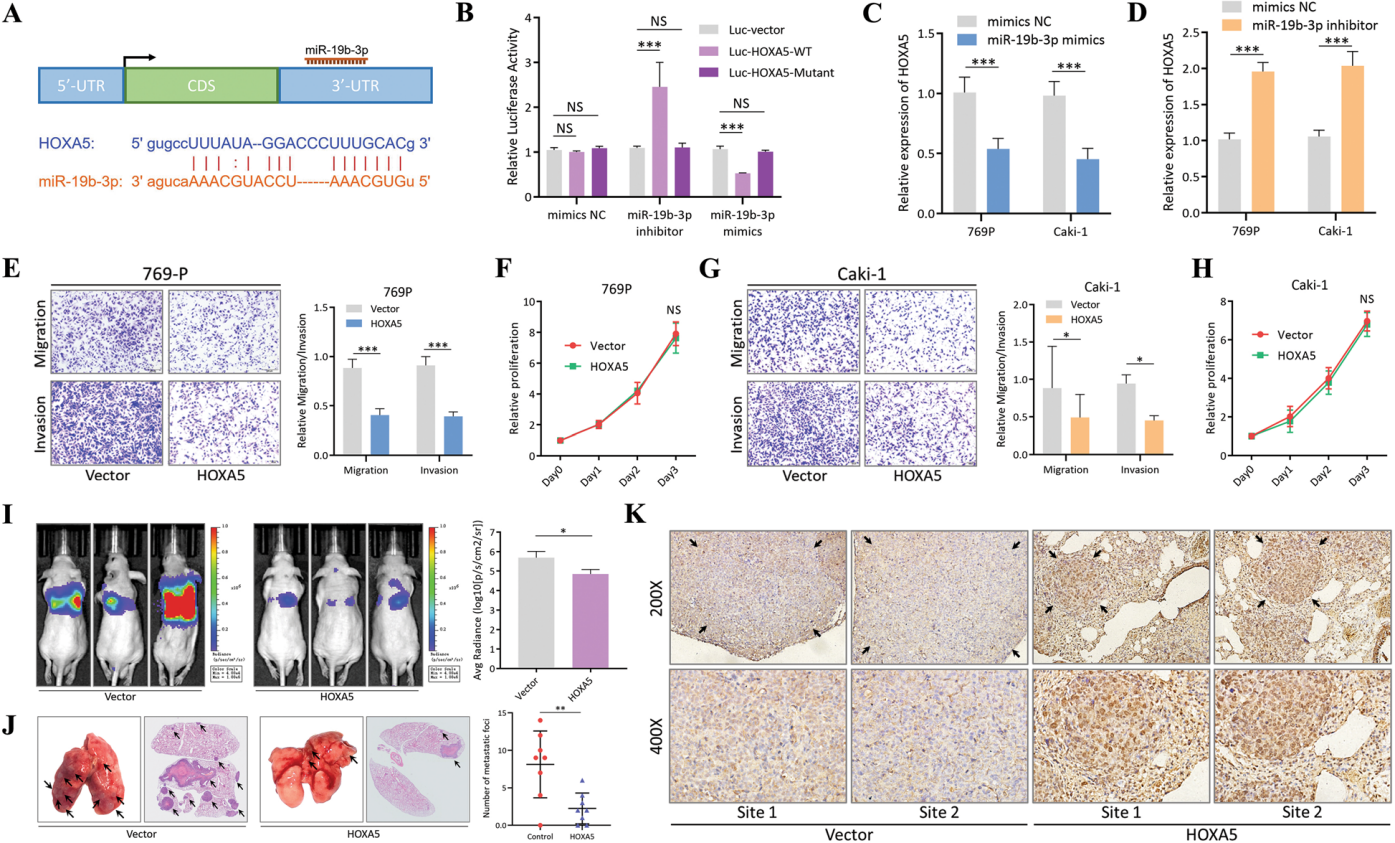
miR-19b-3p regulates the progression of RCC through inhibiting expression of HOXA5 **a**. The binding site and sequence of miR-19b-3p and HOXA5 3′-UTR region. **b**. Luciferase reporter assay with vector, HOXA5 wild-type sequence and HOXA5 mutant sequence transfected miR-19b-3p inhibitor or miR-19b-3p mimics. Vector group was used as normalization control. **c**. qRT-PCR quantification of HOXA5 in 769P and Caki-1 cells transfected with control mimics NC/miR-19b-3p mimics. The mimics NC group was used as normalization. **d**. qRT-PCR quantification of HOXA5 in 769P and Caki-1 cells transfected with control mimics NC/miR-19b-3p inhibitor. The mimics NC group was used as normalization. **e**. Representative images (left) and quantification (right) data of Transwell migration/invasion assay of 769P cells with vector/HOXA5-overexpression. Cell number was determined by counting five random fields under microscope. **f**. Proliferative activity of 769P cells with vector/HOXA5-overexpression measured by CCK8 assay. Levels were normalized to day 0. **g**. Representative images (left) and quantification (right) data of Transwell migration/invasion assay of Caki-1 cells with vector/HOXA5-overexpression. Cell number was determined by counting five random fields under microscope. **h**. Proliferative activity of Caki-1 cells with vector/HOXA5-overexpression measured by CCK8 assay. **i**. Representative images (left) and in vivo luciferase activity quantification (right) data of mouse tail-vein lung metastasis model. 769P cells transfected with vector/ HOXA5-overexpression plasmid were used in the injection. Images were taken 8 weeks after the injection. **j**. Representative HE-stain images (left) and lung-metastasis foci quantification (right) data of 769P mouse tail-vein lung metastasis model. **k**. Representative IHC images of HOXA5 in lung-metastasis foci from 769P vector/HOXA5-overexpression groups. Data are mean ± SD, *n* = 3

In our circNTNG1/miRNA/mRNA interaction network (Fig. 3C), 178 mRNA were predicted to be interact with miR-19b-3p. Among them, 99 mRNAs demonstrated significant downregulation in tumor tissues in TCGA KIRC dataset. Of the top 20 most downregulated mRNAs, only NR3C2, PPARA, SOX6, SMARCA2, HOXA5 and SATB1 were associated with nuclei acid binding activity, which was consistent with the predicted function of miR-19b-3p. After in vitro validation using RCC cell line 769P transfected with a miR-19b-3p mimic, HOXA5 emerged as the most promising downstream target (Additional file 2: Fig. S2F). Thus, it was selected for further investigation. Sequence analysis show that miR-19b-3p could directly bind to the 3′-UTR of HOXA5 mRNA (Fig. 4A). Thus, to further validate the interaction, luciferase reporter plasmids containing wild-type and mutant sequences of HOXA5 were constructed (Additional file 6: Table S1). After co-transfection with either a mimic or an inhibitor of miR-19b-3p, the luciferase activity was altered, indicating there was an interaction (Fig. 4B). The expression level of HOXA5 was also regulated by adding the miR-19b-3p mimic/inhibitor, both on RNA and protein level (Fig. 4C, D and Additional file 2: Fig. S2G, H). To evaluate the effect of HOXA5 on RCC cells, 769P and Caki-1 cells were transfected with a HOXA5 overexpression vector. HOXA5 mRNA and protein were significantly upregulated in these cells compared to controls (Additional file 3: Fig. S3A-C). Overexpression decreased both RCC cells migration and invasion abilities (Fig. 4E, G), without changing the proliferation ability (Fig. 4F, H). Additionally, two HOXA5-targeting siRNAs, which exhibited significant knockdown efficiency in 769P and Caki-1 cells (Additional file 3: Fig. S3D-F), led to increased migration and invasion, but again no change in proliferation (Additional file 3: Fig. S3G-J). Consistent with the in vitro results, the use of HOXA5-overexpressing 769P cells led to decreased pulmonary colonization in the mouse tail vein injection model of metastasis (Fig. 4I). Microscopic examination also demonstrated significantly fewer pulmonary metastatic foci in the HOXA5-overexpressing group compared to the control group (Fig. 4J). IHC staining of lung sections confirmed that higher expression of HOXA5 indeed led to fewer metastatic foci formation (Fig. 4K). Consistently, overexpression of HOXA5 in another cell line 786-O also caused similar in vitro effects, resulting in less pulmonary metastasis (Additional file 3: Fig. S3K-M).

### HOXA5 is a unique tumor suppressor for RCC among HOXA family

**Fig. 5:**
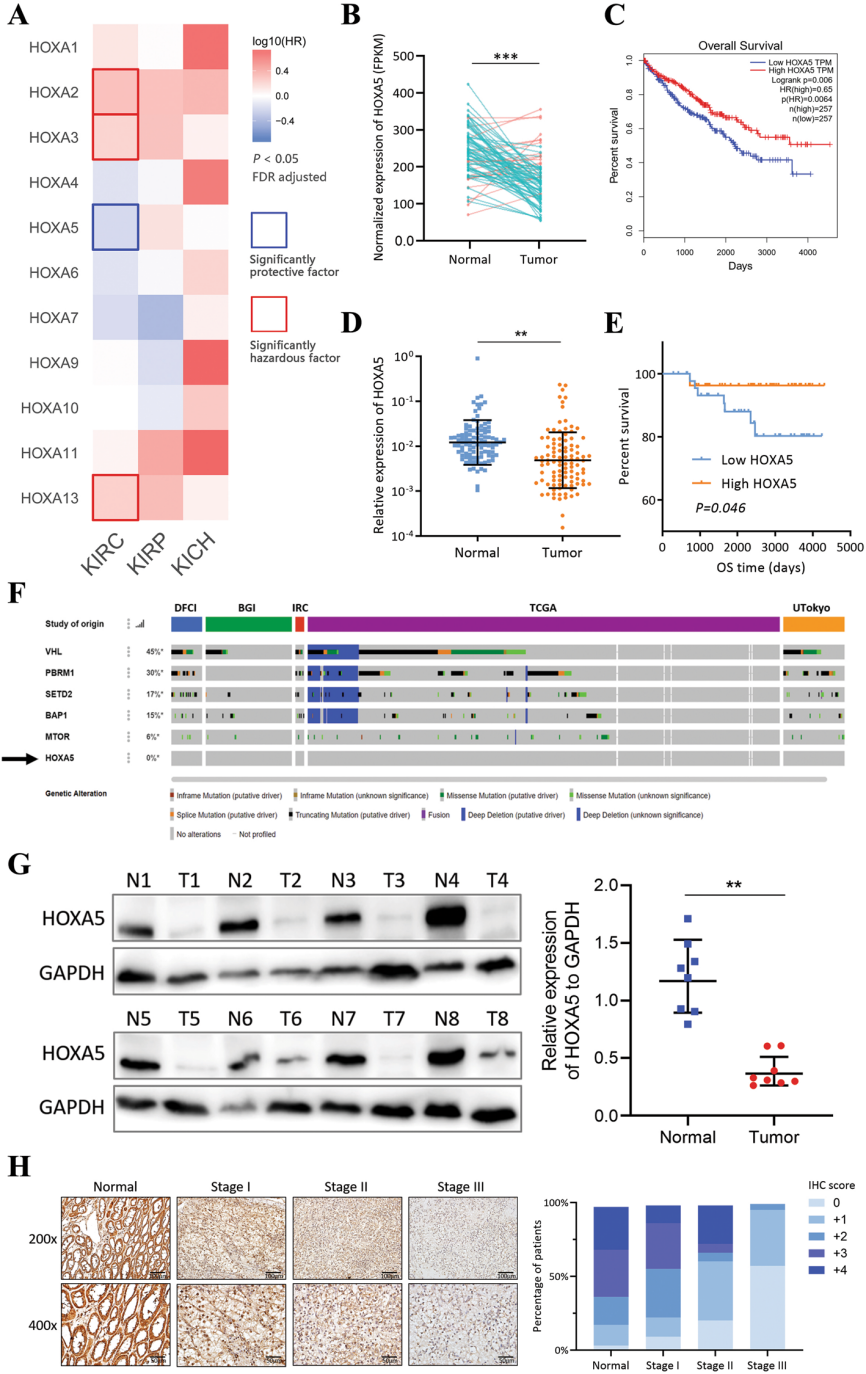
HOXA5 is a unique tumor suppressor for RCC among HOXA family. **a**. Survival profiling of the HOXA family of the KIRC, KIRP and KICH datasets from TCGA. Solid border outline indicates significant survival gene. HR, hazard ratio; FDR, false discovery rate. **b**. Expression analysis of HOXA5 in 72 pairs of match tumors and normal adjacent tissues from KIRC. **c**. Survival analysis (overall survival) of HOXA5 in TCGA KIRC dataset. **d**. Expression analysis of HOXA5 in tumors and normal adjacent tissues in our patient cohort (total 102 patients). **e**. Survival analysis (overall survival) of HOXA5 in our patient cohort. **f**. Genomic alteration rate of HOXA5 and several other frequently mutated genes in five different datasets (DFCI, BGI, IRC, TCGA, and UTokyo). Data was obtained from cBioPortal website. **g**. Immunoblotting analysis of HOXA5 protein expression in 8 pairs of fresh tumors and normal adjacent tissues. GAPDH was used as positive control. **h**. Representative images of HOXA5 IHC staining in patients with different clinical stages (left) and quantification of IHC scores (right)

A survival analysis of HOXA family members was performed on the TCGA RCC datasets. Several HOXA family members (HOXA2, HOXA3, HOXA5, and HOXA13) were associated with the prognosis of the KIRC (ccRCC) subtype. Interestingly, HOXA5 was the only member that correlated with better survival (Fig. 5A). HOXA5 was significantly downregulated in KIRC tissues compared to matched normal tissues (Fig. 5B), and was associated with better patient survival (Fig. 5C). Our own patient cohort also confirmed that HOXA5 was a tumor suppressor, with similar tissue expression pattern and survival result (Fig. 5D, E). Also, in renal tumor tissues with concurrent distant metastasis, the expression of HOXA5 was significantly lower (Additional file 4: Fig. S4A). The expression alteration of HOXA5 was unlikely due to mutation or deletion, since HOXA5 had a 0% mutation and deletion rate across five ccRCC datasets (DFCI, BGI, IRC, TCGA, and UTokyo), compared to several genes with high genetic alterations (VHL, PBRM1, SETD2, BAP1 and MTOR; Fig. 5F), further supporting HOXA5 was post-transcriptionally regulated by non-coding RNA network. Moreover, at the protein level, HOXA5 was lower in tumors compared to the normal adjacent tissues (Fig. 5G), and it decreased with advanced clinical stages (Fig. 5H).

### HOXA5 suppresses epithelial–mesenchymal transition (EMT) activation by downregulating SNAI2 (slug)

**Fig. 6:**
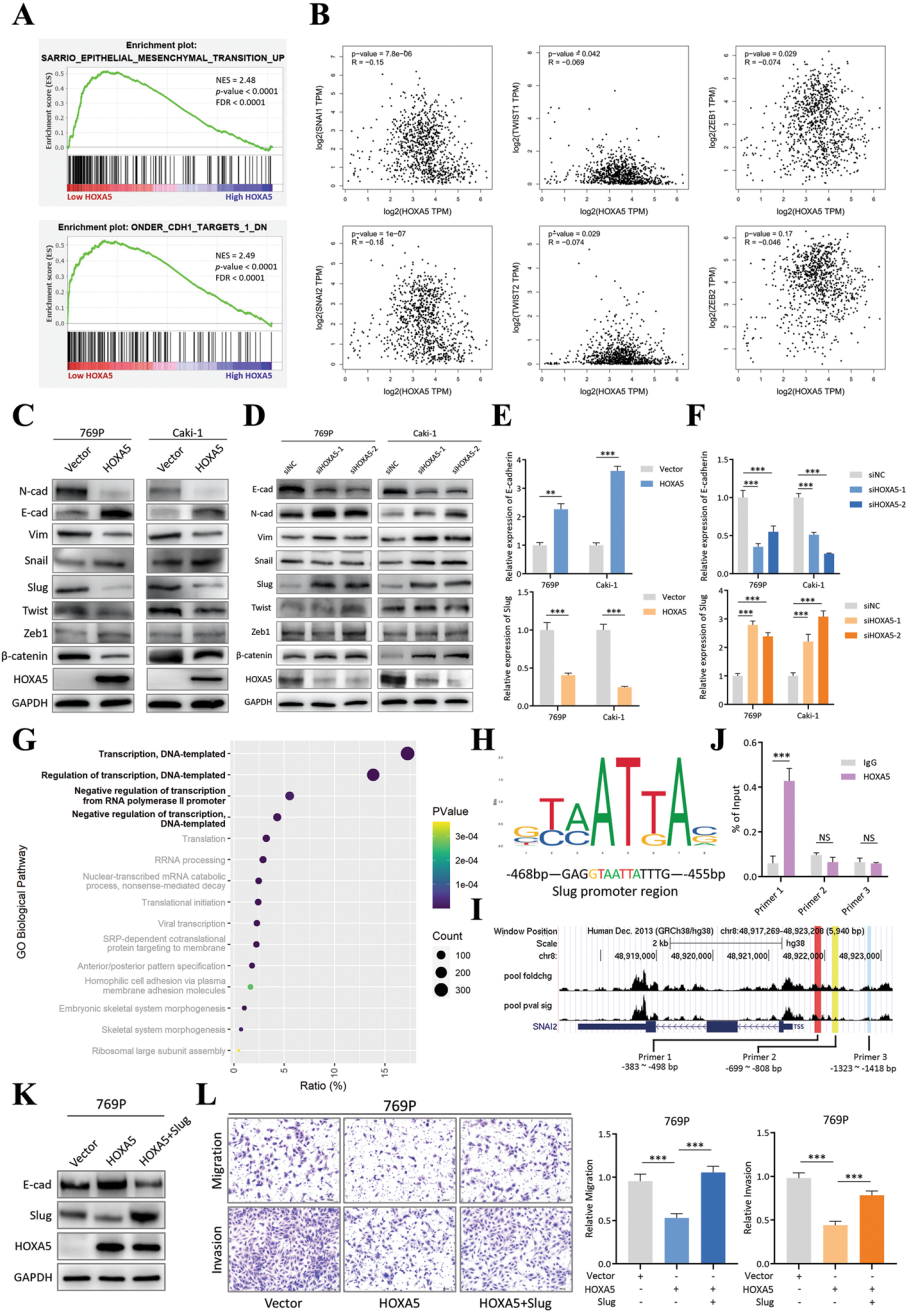
HOXA5 suppressed epithelial–mesenchymal transition (EMT) activation by downregulating SNAI2 (Slug). **a**. GSEA analysis on TCGA KIRC patient cohort (low HOXA5 vs. high HOXA5 patients, median expression as cut-off point). NES, normalized enrichment score; FDR, false discovery rate. **b**. Correlation analysis between HOXA5 and six EMT drivers (Snail, Slug, TWIST1, TWIST2, ZEB1, and ZEB2) in the TCGA KIRC dataset. Data was obtained from GEPIA website. **c**. Immunoblotting of several EMT drivers and markers in 769P and Caki-1 cells with control vector/HOXA5-overexpression. GAPDH was used as normalization control. **d**. Immunoblotting of several EMT drivers and markers in 769P and Caki-1 cells with control siNC/siHOXA5 siRNAs. GAPDH was used as normalization control. **e**. qRT-PCR quantification of E-cadherin and Slug in 769P and Caki-1 cells with control vector/HOXA5-overexpression. The control vector group was used as normalization. **f**. qRT-PCR quantification of E-cadherin and Slug in 769P and Caki-1 cells with control siNC/siHOXA5 siRNAs. The control siNC group was used as normalization. **g**. Bubble plot of GO biological pathway analysis on HOXA5-related genes. *P* value indicated the significance of the reported pathway. Count indicated how many HOXA5-related genes were involved in a particular pathway. Ratio indicated percentage of HOXA5-related genes in a particular pathway within the whole gene list. **h** Motif recognized by HOXA5, predicted by JASPAR transcription factor database (upper). Part of Slug promoter sequence (lower). Colored letters indicated sequence matching the HOXA5 motif. **i**. ChIP-seq dataset (GSE170384) visualization and primer design. **j**. ChIP-qPCR result of DNA fragments pulled-down by Flag antibody in Flag-HOXA5 overexpression 769P cells. Non-specific IgG was used as control. Primer 1, 2 and 3 were specific to different region of Slug promoter. **k**. Immunoblotting of HOXA5-Slug salvage experiment. 769P cells were first transfected with HOXA5-overexpression plasmid, followed by Slug-overexpression plasmid. GAPDH was used as normalization control. **l**. Transwell assay of the 769P HOXA5-Slug salvage experiment. Representative images (left) and quantification (right) data were showed. Data are mean ± SD, *n* = 3

To determine the downstream pathway regulated by HOXA5, GSEA was performed. Using the TCGA KIRC (ccRCC) dataset, the inactivation of EMT pathway was shown to be enriched in the high HOXA5 expression group (Fig. 6A). Since the EMT pathway is an important regulator of tumor progression [26], we hypothesized that HOXA5 inhibited RCC metastasis by suppressing this pathway. To further explore, expression correlation analyses between HOXA5 and several well-recognized EMT drivers, including SNAI1 (Snail), SNAI2 (Slug), TWIST1, TWIST2, ZEB1, and ZEB2 (Fig. 6B), were performed. Only the Snail/Slug family was significantly negatively correlated with HOXA5. The other drivers either had marginal significance or a weak magnitude of correlation. In order to test this result, the expression levels of these EMT drivers and several EMT markers were checked. In two HOXA5-overexpressing RCC cell lines, the epithelial marker E-cadherin was upregulated, while the mesenchymal markers N-cadherin and vimentin were downregulated. Among several EMT drivers, Slug demonstrated more pronounced alteration than Snail, Twist or Zeb1 (Fig. 6C, E). When HOXA5 was knocked down by siRNA, these changes all reverted (Fig. 6D, F). These results indicated that HOXA5, as a tumor suppressor in RCC, may exert its function by inhibiting the EMT driver Slug, which in turn inhibited metastasis.

However, the exact mechanism underlying how HOXA5 inhibited Slug remained unrevealed. HOXA5 is reported to be a canonical DNA-binding protein, and GO analysis confirmed this, as the top four HOXA5-regulated pathways were associated with transcriptional activity (Fig. 6G). As such, HOXA5 may act as a transcriptional suppressor to achieve its inhibitory role on Slug. Combining the JASPAR database and sequence analysis, we discovered that the promoter region of Slug contained a specific sequence that was recognized by HOXA5(Fig. 6H). Also, referring a HOXA5 ChIP-seq dataset in liver cancer (GSE170384), several peaks were identified in the Slug promoter region (Fig. 6I). Based on this, three primer sets targeting the Slug promoter region were designed (primer 1: − 383 to − 498 bp, primer 2: − 699 to − 808 bp, primer 3: − 1323 to − 1418 bp). Only primer 1 covered the HOXA5-binding site (Fig. 6I). As expected, only primer 1 was significantly enriched in the HOXA5 ChIP-qPCR experiment (Fig. 6J). The HOXA5/Slug/EMT relationship was further supported by a salvage experiment in which protein and cell migration/invasion abilities exhibited corresponding changes with Slug re-expression after HOXA5 overexpression (Fig. 6K, L). These results confirmed that HOXA5 bound to the promoter region of Slug and inhibited its expression, thereby regulating EMT.

### HOXA5 recruits DNMT3A to increase the DNA methylation level in the promoter region of slug

**Fig. 7:**
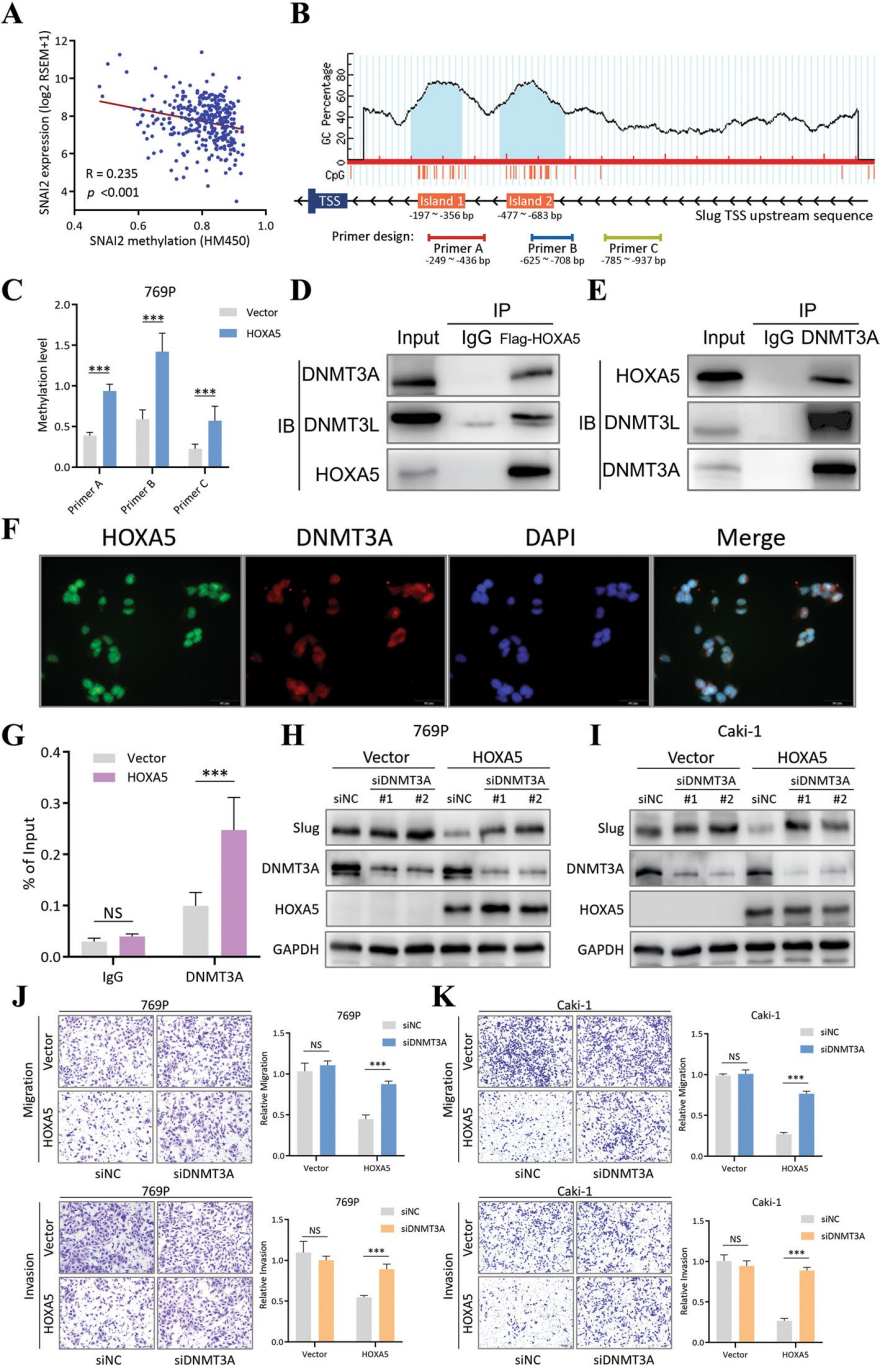
HOXA5 recruited DNMT3A to increase the DNA methylation level in the promoter region of Slug. **a**. Correlation between Slug methylation and expression from the cBioPortal website. **b**. CpG island analysis of the promoter region of Slug and MeDIP primer design. TSS, transcription start site. **c**. MeDIP assay on 769P cells with vector/HOXA5-overexpression conditions. Primer A, B and C were specific to different region of the Slug promoter. **d**. Immunoprecipitation using Flag antibody in Flag-HOXA5 overexpression 769P cells. Non-specific IgG was used as negative control. **e**. Immunoprecipitation using DNMT3A antibody in Flag-HOXA5 overexpression 769P cells. Non-specific IgG was used as negative control. **f**. Immunofluorescence co-localization of HOXA5 and DNMT3A. DAPI was used to indicate the location of nuclei. **g**. ChIP-qPCR assay using a DNMT3A antibody on 769P cells with vector/HOXA5-overexpression conditions. Non-specific IgG was used as control. **h**. Immunoblotting of HOXA5-DNMT3A salvage experiment. 769P cells were transfected with DNMT3A siRNAs with or without Flag-HOXA5 overexpression. GAPDH was used as normalization control. **i**. Immunoblotting of HOXA5-DNMT3A salvage experiment. Caki-1 cells were transfected with DNMT3A siRNAs with or without Flag-HOXA5 overexpression. GAPDH was used as normalization control. **j**. Transwell assay of the 769P HOXA5-DNMT3A salvage experiment. Representative images (left) and quantification (right) data were showed. **k**. Transwell assay of the Caki-1 HOXA5-DNMT3A salvage experiment. Representative images (left) and quantification (right) data were showed. Data are mean ± SD, *n* = 3

After identifying the negative relationship between HOXA5 and Slug, the interaction between HOXA5 and the Slug promoter sequence was further investigated. Using cBioPortal, we found a negative correlation between Slug mRNA expression and its promoter methylation level (Fig. 7A). Also, within the Slug promoter region, two CpG islands were predicted (island 1: − 197 to − 356 bp, island 2: − 477 to − 683 bp, Fig. 7B), which were close to the HOXA5-binding site. Thus, we hypothesized that HOXA5 may inhibit Slug expression by increasing the methylation level of its promoter region. To confirm this, three sets of primers were designed (primer A: − 249 to − 436 bp, primer B: − 625 to − 708 bp, primer C: − 785 to − 937 bp), among which primers A and B covered islands 1 and 2, respectively (Fig. 7B). By performing MeDIP on 769P cells before and after HOXA5 overexpression, the methylation level was found to be increased in all three sites, and a more pronounced enrichment was detected by primers A and B (Fig. 7C). Using another cell line Caki-1, a similar result was observed (Additional file 4: Fig. S4B). In human cells, there are three major DNA methylation writers: DNMT3A, DNMT3B and DNMT1 [27]. We wondered if HOXA5 could couple with any of these writers, resulting the methylation level alteration within the Slug promoter. By performing immunoprecipitation, a significant amount of DNMT3A was pulled down along with HOXA5, compared to DNMT3B and DNMT1, which showed minimal enrichment after pull-down (Fig. 7D and Additional file 4: Fig. S4C). Also, DNMT3L, which serves as an enhancer and stabilizer protein for DNMT3A, was pulled down along with DNMT3A (Fig. 7D). Similarly, HOXA5 was pulled down along with DNMT3A (Fig. 7E). IF co-localization confirmed that HOXA5 and DNMT3A had similar subcellular distribution in the nucleus (Fig. 7F), suggesting an interaction. Furthermore, ChIP-qPCR using the primer covering the HOXA5-binding site (primer 1: − 383 to 498 bp) demonstrated significant increased DNMT3A recruitment to the Slug promoter region after HOXA5 overexpression (Fig. 7G). Salvage experiments also confirmed the indispensable role of DNMT3A in HOXA5-mediated Slug and EMT inhibition. The Slug expression was disinhibited and migration/invasion abilities of RCC cells were recovered after simultaneous HOXA5 overexpression and DNMT3A knockdown (Fig. 7H-K). These results indicated that HOXA5 inhibited Slug expression by recruiting DNMT3A to form a complex and subsequently increasing the methylation level of the Slug promoter.

### Integrity of the circNTNG1/miR-19b-3p/HOXA5 regulatory axis

**Fig. 8:**
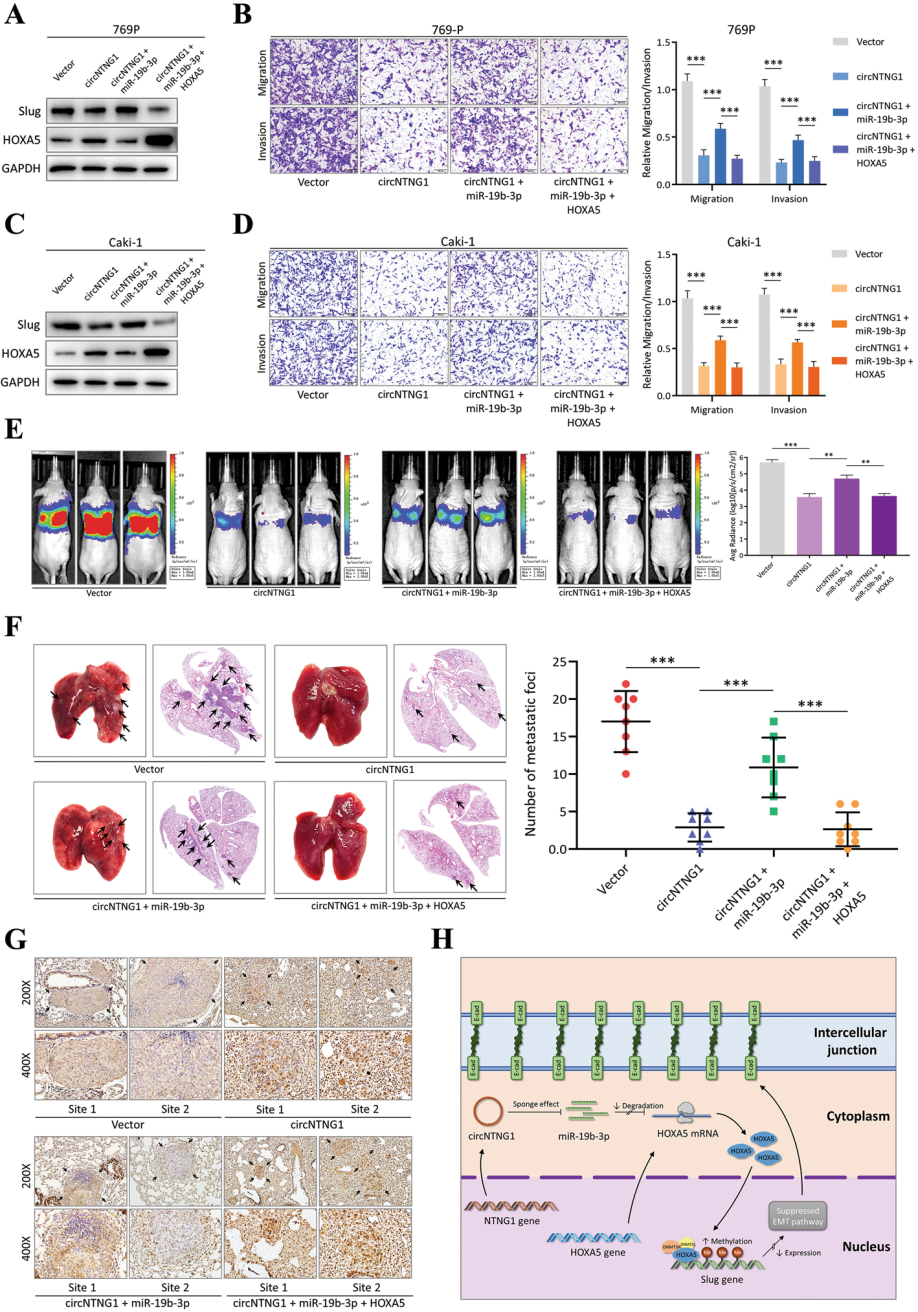
Integrity of the circNTNG1/miR-19b-3p/HOXA5 regulatory axis. **a**. Immunoblotting of HOXA5 and Slug in 769P cells circNTNG1 overexpression salvage experiment. Cells were treated with circNTNG1 overexpression with/without miR-19b-3p mimics, and further salvaged with HOXA5 overexpression. GAPDH was used as positive control. **b**. Representative images (left) and quantification (right) data of Transwell migration/invasion assay of 769P cells circNTNG1 overexpression salvage experiment. **c.** Immunoblotting of HOXA5 and Slug in Caki-1 cells circNTNG1 overexpression salvage experiment. Cells were treated with circNTNG1 overexpression with/without miR-19b-3p mimics, and further salvaged with HOXA5 overexpression. GAPDH was used as positive control. **d**. Representative images (left) and quantification (right) data of Transwell migration/invasion assay of Caki-1 cells circNTNG1 overexpression salvage experiment. **e**. Representative images (left) and in vivo luciferase activity quantification (right) data of mouse tail-vein lung metastasis model. 769P cells with vector, circNTNG1-overexpression, circNTNG1-overexpression+miR-19b-3p overexpression or circNTNG1-overexpression+miR-19b-3p overexpression+HOXA5 overexpression were used in the injection. Images were taken 8 weeks after the injection. **f**. Representative HE-stain images (left) and lung-metastasis foci quantification (right) data of 769P mouse tail-vein lung metastasis model. **g**. Representative IHC images of HOXA5 in lung-metastasis foci from 769P mouse tail-vein lung metastasis model. **h**. Schematic diagram of the intact circNTNG1/miR-19b-3p/HOXA5 pathway in controlling the Slug expression and EMT activity via methylation change. Data are mean ± SD, *n* = 3

In our patient cohort, members of the circNTNG1/miR-19b-3p/HOXA5 regulatory axis were closed correlated (Additional file 5: Fig. S5A-C). To further investigate the integrity of this axis, salvage experiments were performed. After applying a mimic or an inhibitor of miR-19b-3p, the expression levels of HOXA5, Slug, and EMT markers demonstrated corresponding changes (Additional file 5: Fig. S5D, E). Further, adding a miR-19b-3p mimic after circNTNG1 overexpression reversed the change of HOXA5 and Slug protein expression (Fig. 8A, C and Additional file 5: Fig. S5F), as well as the migration and invasion phenotype of RCC cells (Fig. 8B, D and Additional file 5: Fig. S5G). These changes were again salvaged by transfecting cells with HOXA5 overexpressing vector (Fig. 8A-D and Additional file 5: Fig. S5F, G). Consistently, in mouse tail vein injection model of metastasis, co-expression of miR-19b-3p could abolish the tumor-inhibitive effect of circNTNG1 overexpression in 769P and 786-O cells, leading to recovery of pulmonary colonization ability (Fig. 8E and Additional file 5: Fig. S5H). Again, if cells were further transfected with HOXA5 overexpressing vector, the effect of miR-19b-3p was suppressed (Fig. 8E and Additional file 5: Fig. S5H). The pulmonary metastatic foci were also confirmed by microscopic examination (Fig. 8F and Additional file 5: Fig. S5I). IHC staining confirmed that lung sections with fewer metastatic foci demonstrated higher HOXA5 expression (Fig. 8G and Additional file 5: Fig. S5J). These findings indicate that the circNTNG1/miR-19b-3p/HOXA5 axis exists in RCC and regulates RCC progression (Fig. 8H).

## Discussion

Over the past two decades, the incidence of RCC has been increasing [1]. This can be partially attributed to the increase in routine imaging for many other disorders, because a considerable portion of newly diagnosed RCC cases are discovered incidentally while evaluating other diseases [28]. Despite the rising incidence, the overall survival of RCC patients has also substantially improved [1]. The oncogenic mechanisms and therapeutic targets of RCC have been studied extensively and several genes, including VHL, PBRM1, BAP1, and SETD2, were successfully identified as being critical in the development and treatment of RCC [28]. However, the overall understanding of RCC remains limited, and more effort should be made to establish deeper insights, especially for metastatic RCC.

CircRNAs were once considered as errors during cell transcription, resulting from defective splicing, and exerted no biological function [29]. However, over the last several years, thanks to the rapid development of high-throughput sequencing technique, a lot of circRNAs have been identified. Many of them have indispensable function in normal and cancer cells [5–7]. In our study, we also applied circRNA sequencing to explore the significant circRNAs in RCC. After filtering the circRNAs located on frequently lost chromosome regions in RCC [15–17], and validating with external datasets, we managed to discover a circRNA with significant tumor-suppressing function in RCC: circNTNG1. Subsequent phenotype and mechanism experiments confirmed that circNTNG1 inhibited the metastasis of RCC via the circNTNG1/miR-19b-3p/HOXA5/EMT pathway. To our knowledge, circNTNG1 and its function have never been studied in RCC before, thus our research provides novel insights of how circRNA regulates the progression of RCC with solid experimental evidences.

The predicted functions of miR-19b-3p were ion binding and nucleic acid binding. In this study, it is confirmed that miR-19b-3p can regulated HOXA5 to bind to the promoter region of Slug, which directly controls the invasion and migration of RCC cells via EMT process. However, as for its ion binding function, and how it controls the aggressiveness of RCC, has not been fully elucidated. In normal cells, miR-19b-3p is found to be playing an important role in ion metabolism. For example, miR-19b-3p can regulate the potassium channel in the brain of rats with post-traumatic epilepsy, leading to the onset of seizure [30]. Also, miR-19b-3p is involved in the differentiation of osteoblast during human embryo development, which requires intense calcium metabolism [31]. These evidences indicate that miR-19b-3p can exert ion binding function under physiological conditions. However, whether or how it controls ion binding in RCC or other cancers require further investigation.

In five ccRCC RNA-seq datasets on cBioPortal, the genomic alteration rate of HOXA5 was 0%, meaning that neither gene mutation nor chromosomal deletion could explain the HOXA5 downregulation. This finding further strengthened the idea that HOXA5 alteration was mediated by a posttranscriptional event, i.e., RNA-level alteration. On the contrary, as circNTNG1 is located in a frequently deleted chromosomal region, the loss of its expression could be explained by DNA-level alteration. Therefore, the low HOXA5 expression may be fundamentally caused by the deletion of circNTNG1 in RCC. However, it is possible that other mechanisms could also regulate HOXA5, and more research is needed to explore this.

We also identified HOXA5 as a novel tumor suppressor in RCC. Its expression was associated with better survival of RCC patients according to both the TCGA datasets and our cohort. In the HOXA family member survival analysis, only HOXA5 demonstrated a protective effect in KIRC. Several other members, including HOXA2, HOXA3, and HOXA13, were significantly associated with poor prognosis in KIRC. These correlations were partially consistent with a previous study [32]. Surprisingly, none of the HOXA family members demonstrated a significant impact in the KIRP and KICH patients. Although this could represent a true-negative correlation, this could also be attributable to the lower sample sizes in the two datasets (286 in KIRP and 66 in KICH vs. 523 in KIRC). The sample sizes reflected the proportions of the three RCC pathological subtypes, with KIRC (ccRCC) accounting for > 80% of the cases in clinical setting [2]. Certain HOXA family members may be shown to have clinical implications in KIRP and KICH if a larger patient cohort were to be analyzed.

Although HOXA5 was identified as a nucleus-located DNA-binding protein, the modulation of its mRNA by happens in the cytoplasm, via the circNTNG1/miR-19b-3p/HOXA5 (mRNA) axis. The axis alters the quantity of HOXA5 mRNA being translated and thus, eventually alters the quantity of HOXA5 protein translocation into the nucleus. The subsequent GSEA analysis emphasized that loss of HOXA5 may lead to activation of the EMT pathway. Additionally, we found that the Slug gene, which is a potent regulator of the EMT pathway, has a specific HOXA5-binding sequence in its promoter region. EMT, which plays a crucial role during embryogenesis [33], was first described in the 1980s. Cells that undergo EMT lose their epithelial morphology and acquire stronger mobility, facilitating migration, and EMT may also regulate the invasion of cancer cells [34]. Slug, as a transcription factor, is directly involved in controlling EMT activation in RCC cells [35]. Slug can bind to the E-boxes located in the promoter of E-cadherin, which downregulates E-cadherin and thus causes EMT activation [36]. We observed a consistent result. HOXA5 inhibited Slug expression, which in turn caused E-cadherin downregulation. IP, ChIP, and MeDIP assays further confirmed that HOXA5 could recruit DNMT3A/DNMT3L to form a complex, which inhibited Slug expression, thus inactivating the EMT pathway.

In mammalian cells, there are three DNA methyltransferases, comprising DNMT1, DNMT3A, and DNMT3B, which catalyze DNA methylation [27]. DNMT1 preferentially mediates the maintenance of CpG methylation patterns during DNA replication. DNMT3A and DNMT3B are more involved in de novo methylation, controlling a great variety of physiological and pathological processes. Also, DNMT3L, another member of the DNMT3 family, is an important regulator of methylation. DNMT3L itself lacks catalytic function but it can bind to DNMT3A, stimulating DNMT3A catalytic activity and maintaining its stability [37]. In our study, although both DNMT3A and DNMT3B were pulled down in the IP assays, DNMT3A was much more enriched and thus was considered to exert the major effect in the methylation of the Slug promoter.

## Conclusions

Based on our experimental data, in RCC, circNTNG1/miR-19b-3p/HOXA5 axis can regulate the epigenetic silencing of Slug via DNMT3A/DNMT3L methylation writer, thus interfering EMT and metastasis of RCC. Together, our results provide evidence of an intact pathway that regulates RCC progression, along with several potential therapeutic targets for future study.

## Supplementary Information

**Additional file 1 Fig. S1.** The clinical properties of circNTNG1 in RCC. **a**. Workflow of filtering frequently deleted circRNAs in RCC using two online circRNA microarray datasets. Numbers near the arrows in each steps indicated the remaining circRNAs. **b**. Expression analysis of circNTNG1 in different clinical stage in our patient cohort (total 102 patients). **c**. Expression analysis of circNTNG1 in different Fuhrman grade in our patient cohort (total 102 patients). **d**. Expression analysis of circNTNG1 in renal tumors from RCC patients with/without distant metastasis at the time of diagnosis (total 28 patients). **e**. Representative images (left) and quantification (right) data of Transwell migration/invasion assay of 786-O cells with vector/circNTNG1-overexpression. Cell number was determined by counting five random fields under microscope. **f**. Proliferative activity of 786-O cells with vector/circNTNG1-overexpression measured by CCK8 assay. Levels were normalized to day 0. Data are mean ± SD, *n* = 3.

**Additional file 2 Fig. S2** The clinical properties of miR-19b-3p in RCC and its function in RCC cells. **a**. The pathways regulated by miR-19b-3p. Data was obtained from online database mirPath. Count indicated that the number of miRNA-related genes involved in the pathway. **b**. Expression analysis of miR-19b-3p in tumors and normal adjacent tissues in our patient cohort (total 102 patients). **c**. Survival analysis (overall survival) of miR-19b-3p in our patient cohort. **d**. Survival analysis (recurrent-free survival) of miR-19b-3p in our patient cohort. **e**. Expression analysis of miR-19b-3p in renal tumors from RCC patients with/without distant metastasis at the time of diagnosis (total 28 patients). **f**. qRT-PCR screening of 6 candidate mRNAs in 769P cells treated with mimics NC/miR-19b-3p mimics. Mimics NC group was used as normalization control. **g.** Immunoblotting of HOXA5 in 769P cells with treatment of miR-19b-3p mimics or miR-19b-3p inhibitor. GAPDH was used as internal control. **h.** Immunoblotting of HOXA5 in Caki-1 cells with treatment of miR-19b-3p mimics or miR-19b-3p inhibitor. GAPDH was used as internal control. Data are mean ± SD, *n* = 3.

**Additional file 3 Fig. S3** HOXA5 inhibited the migration and invasion of RCC cells. **a**. qRT-PCR quantification of HOXA5 in 769P and Caki-1 cells transfected with control vector/HOXA5-overexpression. The control vector group was used as normalization. **b**. Immunoblotting confirmation of HOXA5 overexpression in 769P. GAPDH was used as normalization control. **c**. Immunoblotting confirmation of HOXA5 overexpression in Caki-1. GAPDH was used as normalization control. **d**. qRT-PCR quantification of HOXA5 in 769P/Caki-1 cells treated with control siNC/siHOXA5 siRNAs. The siNC group was used as normalization. **e**. Immunoblotting confirmation of HOXA5 knockdown in 769P. GAPDH was used as normalization control. **f**. Immunoblotting confirmation of HOXA5 knockdown in Caki-1. GAPDH was used as normalization control. **g**. Representative images (left) and quantification (right) data of Transwell migration/invasion assay of 769P cells treated with control siNC/siHOXA5 siRNAs. **h**. Proliferative activity of 769P cells treated with control siNC/siHOXA5 siRNAs measured by CCK8 assay. **i**. Representative images (left) and quantification (right) data of Transwell migration/invasion assay of Caki-1 cells treated with control siNC/siHOXA5 siRNAs. **j**. Proliferative activity of Caki-1 cells treated with control siNC/siHOXA5 siRNAs measured by CCK8 assay. **k**. Representative images (left) and in vivo luciferase activity quantification (right) data of mouse tail-vein lung metastasis model. 786-O cells transfected with vector/HOXA5-overexpression plasmid were used in the injection. Images were taken 8 weeks after the injection. **l**. Representative HE-stain images (left) and lung-metastasis foci quantification (right) data of 786-O mouse tail-vein lung metastasis model. **m**. Representative IHC images of HOXA5 in lung-metastasis foci from 786-O vector/HOXA5-overexpression group. Data are mean ± SD, *n* = 3.

**Additional file 4 Fig. S4** HOXA5 recruited DNMT3A to increase the DNA methylation level in the promoter region of Slug. **a**. Expression analysis of HOXA5 in renal tumors from RCC patients with/without distant metastasis at the time of diagnosis (total 28 patients). **b**. MeDIP assay on Caki-1 cells with vector/HOXA5-overexpression conditions. Primer A, B and C were specific to different region of the Slug promoter. **c**. Immunoprecipitation using Flag antibody in Flag-HOXA5 overexpression cells. Non-specific IgG was used as negative control. Data are mean ± SD, *n* = 3.

**Additional file 5 Fig. S5** The regulation of RCC by circNTNG1/miR-19b-3p/HOXA5 axis. **a**. Correlation analysis of circNTNG1 and miR-19b-3p in our own patient cohort (102 patients). **b**. Correlation analysis of miR-19b-3p and HOXA5 in our own patient cohort (102 patients). **c**. Correlation analysis of HOXA5 and Slug in our own patient cohort (102 patients). **d**. Immunoblotting of HOXA5, Slug and EMT markers with treatment of miR-19b-3p mimics or miR-19b-3p inhibitor in 769P cells. GAPDH was used as internal control. **e**. Immunoblotting of HOXA5, Slug and EMT markers with treatment of miR-19b-3p mimics or miR-19b-3p inhibitor in Caki-1 cells. GAPDH was used as positive control. **f**. Immunoblotting of HOXA5 and Slug in 786-O cells circNTNG1 overexpression salvage experiment. Cells were treated with circNTNG1 overexpression with/without miR-19b-3p mimics, and further salvaged with HOXA5 overexpression. GAPDH was used as positive control. **g**. Representative images (left) and quantification (right) data of Transwell migration/invasion assay of 786-O cells circNTNG1 overexpression salvage experiment. **h**. Representative images (left) and in vivo luciferase activity quantification (right) data of mouse tail-vein lung metastasis model. 786-O cells with vector, circNTNG1-overexpression, circNTNG1-overexpression+miR-19b-3p overexpression or circNTNG1-overexpression+miR-19b-3p overexpression+HOXA5 overexpression were used in the injection. Images were taken 8 weeks after the injection. **i**. Representative HE-stain images (left) and lung-metastasis foci quantification (right) data of 786-O mouse tail-vein lung metastasis model. **j**. Representative IHC images of HOXA5 in lung-metastasis foci from 786-O mouse tail-vein lung metastasis model. Data are mean ± SD, *n* = 3.

**Additional file 6 Table S1** Primers and DNA/RNA sequence used in this study.

**Additional file 7 Table S2** Clinical information of patient samples in the circRNA-seq.

**Additional file 8 Table S3** Frequently Deleted Chromosomes in RCC.

**Additional file 9 Table S4** Significant circRNAs after filtration in our own dataset.

**Additional file 10 Table S5** Significant circRNAs after filtration in two public datasets (GSE137836 and GSE100186).

**Additional file 11 Supplemental materials and methods** Supplemental materials and methods.

## Data Availability

For all data requests, please contact the corresponding author.

## Abbreviations

RCC: Renal cell carcinoma
circRNAs: Circular RNAs
HOXA5: Homeobox A5
TCGA: The Cancer Genome Atlas
Slug: SNAI2
EMT: Epithelial–mesenchymal transition
ccRCC: Clear cell renal cell carcinoma
ncRNA: Non-coding RNA
mRNA: Messenger RNA
KIRC: Kidney renal clear cell carcinoma
KIRP: Kidney renal papillary cell carcinoma
KICH: Kidney chromophobe
GEO: Gene Expression Omnibus
GO: Gene ontology
GSEA: Gene set enrichment analysis
gDNA: Genomic DNA
CCK-8: Cell Counting Kit-8
IP: Immunoprecipitation
ChIP: Chromatin immunoprecipitation
MeDIP: Methylated DNA immunoprecipitation
IF: Immunofluorescence
FISH: Fluorescence in situ hybridization
RIP: RNA pull-down
