# Correction: CircNTNG1 inhibits renal cell carcinoma progression via HOXA5-mediated epigenetic silencing of Slug

**DOI:** 10.1186/s12943-023-01726-w

**Published:** 2023-01-20

**Authors:** Yanping Liang, Junjie Cen, Yong Huang, Yong Fang, Yunfei Wang, Guannan Shu, Yihui Pan, Kangbo Huang, Jiaqi Dong, Mi Zhou, Yi Xu, Junhang Luo, Min Liu, Jiaxing Zhang

**Affiliations:** 1 Department of Laboratory Medicine, The First Affiliated Hospital, Sun Yat-sen University, Guangzhou, 510080 China; 2 Department of Urology, The First Affiliated Hospital, Sun Yat-sen University, Guangzhou, 510080 China; 3 Department of Emergency, The First Affiliated Hospital, Sun Yat-sen University, Guangzhou, 510080 China; 4 Department of Oncology, The First Affiliated Hospital, Sun Yat-sen University, Guangzhou, 510080 China; 5 Department of Urology, Sun Yat-sen University Cancer Center, Guangzhou, 510060 China; 6 Institute of Precision Medicine, The First Affiliated Hospital, Sun Yat-sen University, Guangzhou, 510080 China

**Correction: Mol Cancer 21, 224 (2022)**

**https://doi.org/10.1186/s12943-022-01694-7**

Following the publication of the original article [1], the authors reported that the Additional file 5 is outdated. During the proof process, a mistake due to figure compilation was noticed in Additional file 5 Fig. S5J. The updated version has been provided in this erratum article, please see Additional file section. The authors would like to apologize for this negligence. It does not affect any of the interpretations or conclusions of the article.

## Supplementary Information

**Additional file 5: Fig. S5.** The regulation of RCC by circNTNG1/miR-19b-3p/HOXA5 axis. **A**. Correlation analysis of circNTNG1 and miR-19b-3p in our own patient cohort (102 patients). **B**. Correlation analysis of miR-19b-3p and HOXA5 in our own patient cohort (102 patients). **C**. Correlation analysis of HOXA5 and Slug in our own patient cohort (102 patients). **D**. Immunoblotting of HOXA5, Slug and EMT markers with treatment of miR-19b-3p mimics or miR-19b-3p inhibitor in 769P cells. GAPDH was used as internal control. **E**. Immunoblotting of HOXA5, Slug and EMT markers with treatment of miR-19b-3p mimics or miR-19b-3p inhibitor in Caki-1 cells. GAPDH was used as positive control. **F**. Immunoblotting of HOXA5 and Slug in 786-O cells circNTNG1 overexpression salvage experiment. Cells were treated with circNTNG1 overexpression with/without miR-19b-3p mimics, and further salvaged with HOXA5 overexpression. GAPDH was used as positive control. **G**. Representative images (left) and quantification (right) data of Transwell migration/invasion assay of 786-O cells circNTNG1 overexpression salvage experiment. **H**. Representative images (left) and in vivo luciferase activity quantification (right) data of mouse tail-vein lung metastasis model. 786-O cells with vector, circNTNG1-overexpression, circNTNG1-overexpression+miR-19b-3p overexpression or circNTNG1-overexpression+miR-19b-3p overexpression+HOXA5 overexpression were used in the injection. Images were taken 8 weeks after the injection. **I**. Representative HE-stain images (left) and lung-metastasis foci quantification (right) data of 786-O mouse tail-vein lung metastasis model. **J**. Representative IHC images of HOXA5 in lung-metastasis foci from 786-O mouse tail-vein lung metastasis model. Data are mean ± SD, *n* = 3.
