# Supplementary figures and images for: CircNTNG1 inhibits renal cell carcinoma progression via HOXA5-mediated epigenetic silencing of Slug
Source: Mol Cancer. 2022 Dec 19;21:224. doi: 10.1186/s12943-022-01694-7 (PMC9761964; doi:10.1186/s12943-022-01694-7)

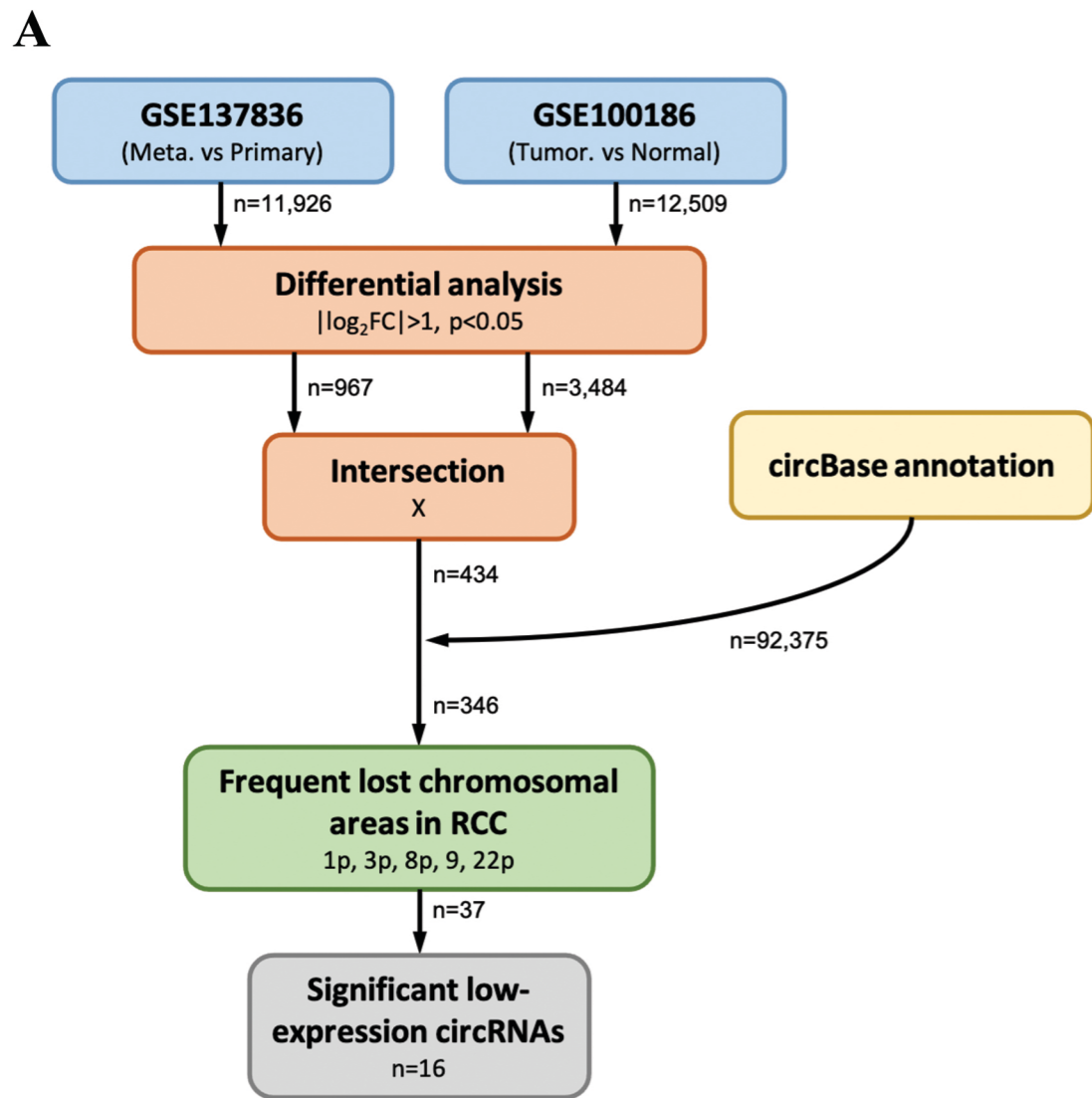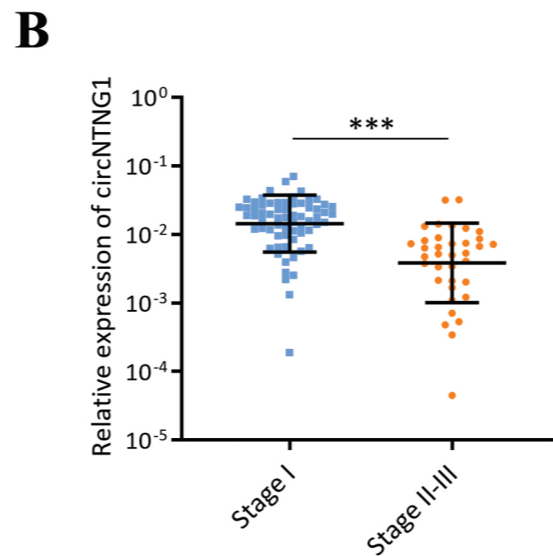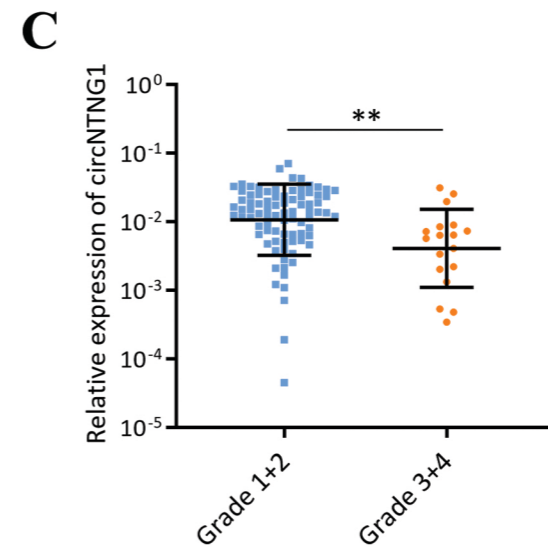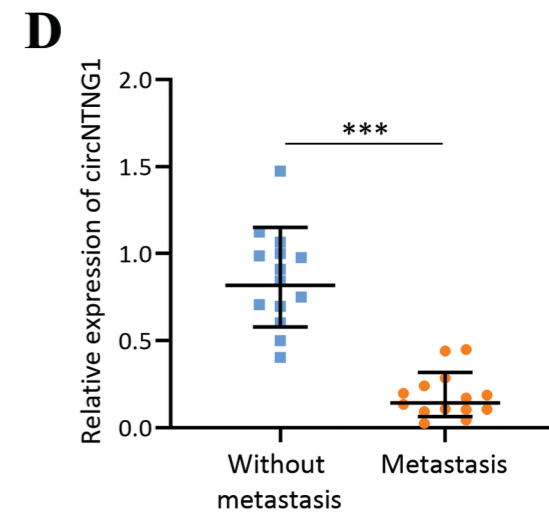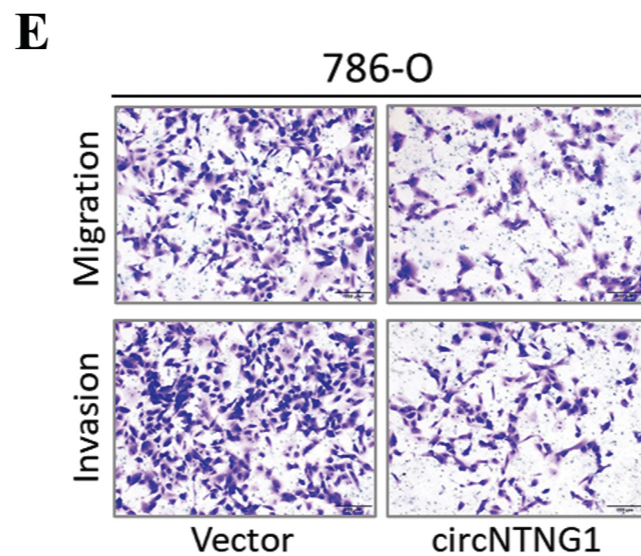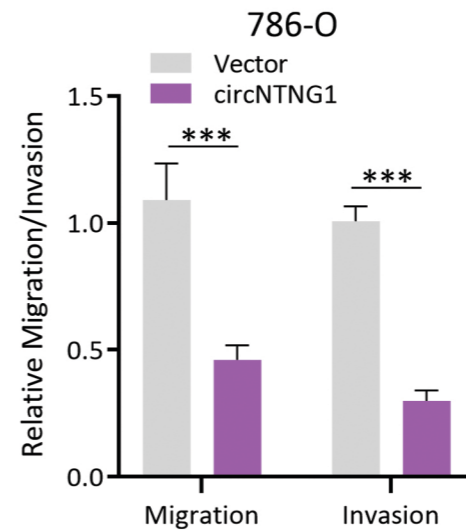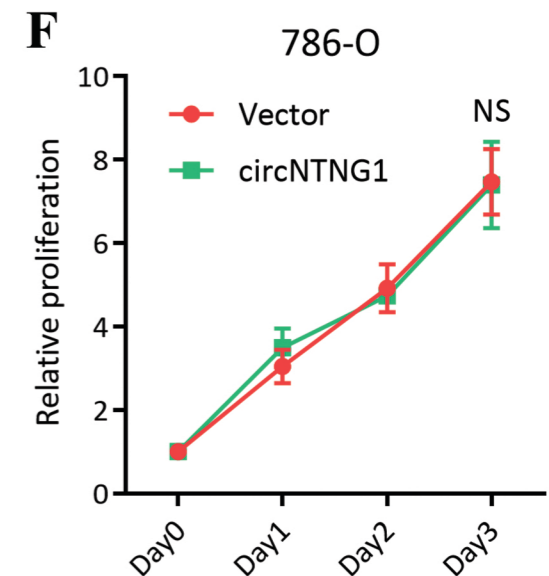

Supplement: Supplementary file 1 — Additional file 1 Fig. S1. The clinical properties of circNTNG1 in RCC. a. Workflow of filtering frequently deleted circRNAs in RCC using two online circRNA microarray datasets. Numbers near the arrows in each steps indicated the remaining circRNAs. b. Expression analysis of circNTNG1 in different clinical stage in our patient cohort (total 102 patients). c. Expression analysis of circNTNG1 in different Fuhrman grade in our patient cohort (total 102 patients). d. Expression analysis of circNTNG1 in renal tumors from RCC patients with/without distant metastasis at the time of diagnosis (total 28 patients). e. Representative images (left) and quantification (right) data of Transwell migration/invasion assay of 786-O cells with vector/circNTNG1-overexpression. Cell number was determined by counting five random fields under microscope. f. Proliferative activity of 786-O cells with vector/circNTNG1-overexpression measured by CCK8 assay. Levels were normalized to day 0. Data are mean ± SD, n = 3. [file 12943_2022_1694_MOESM1_ESM.pdf]

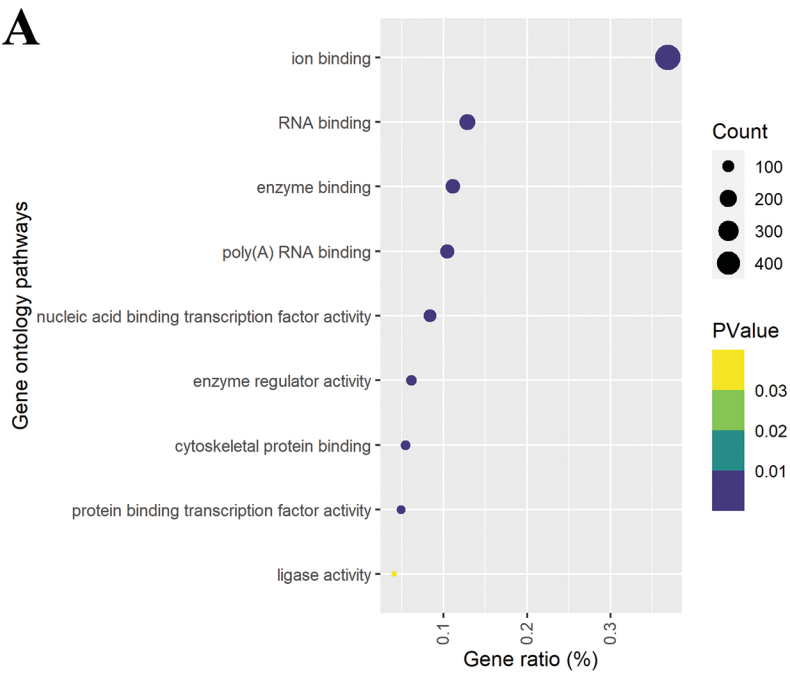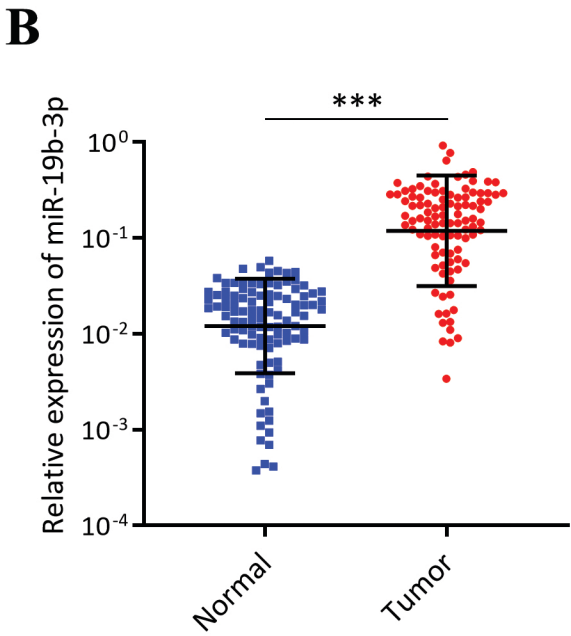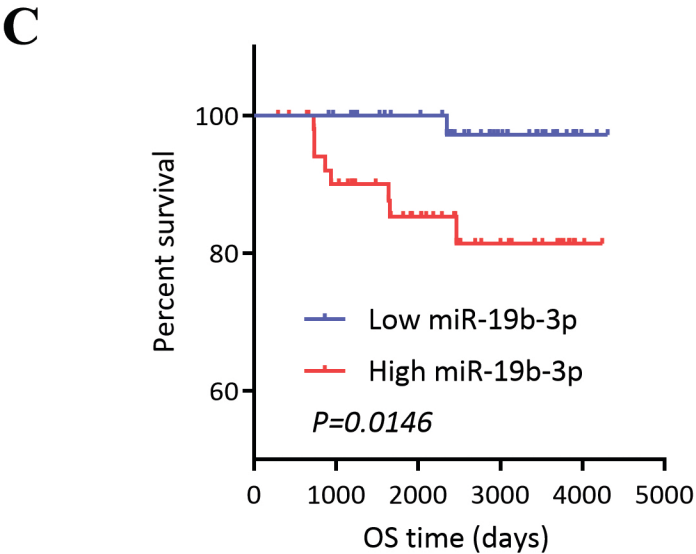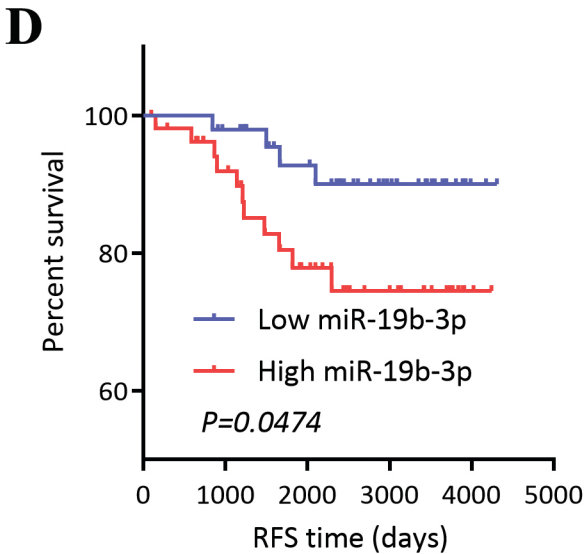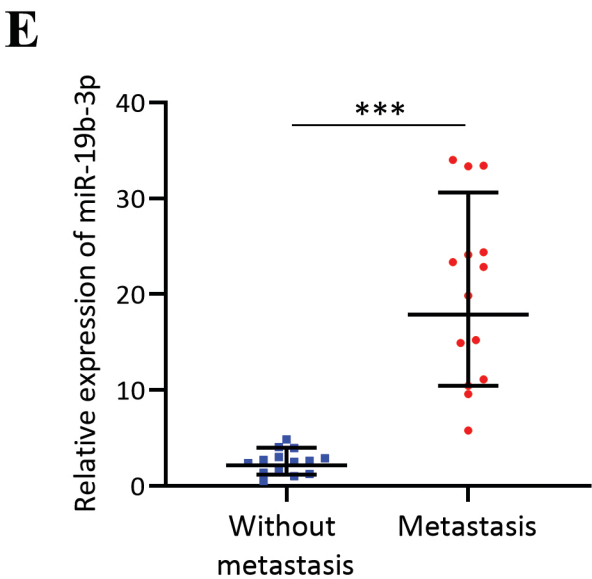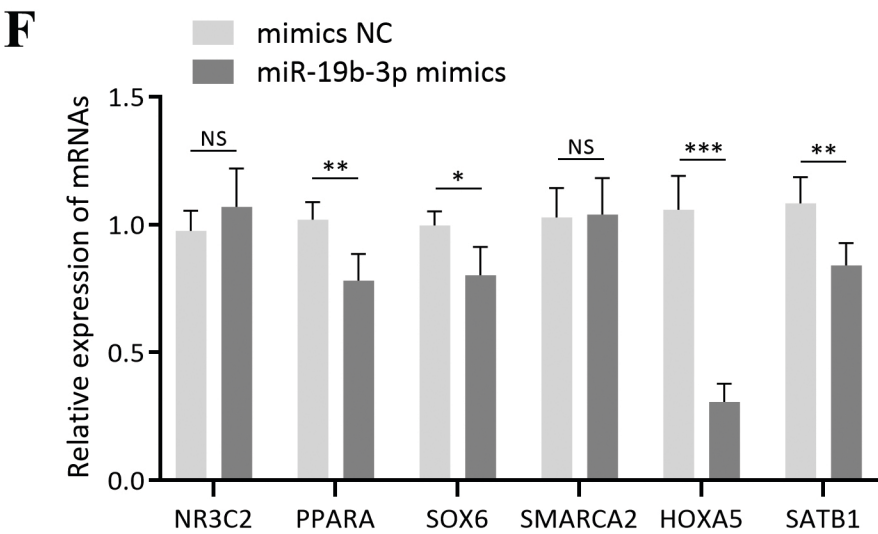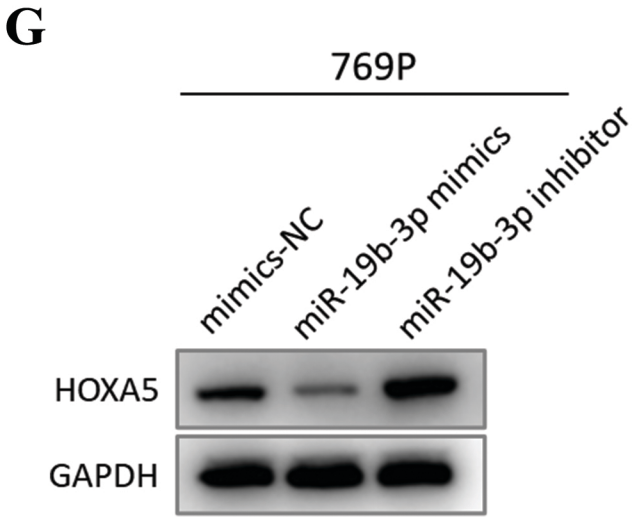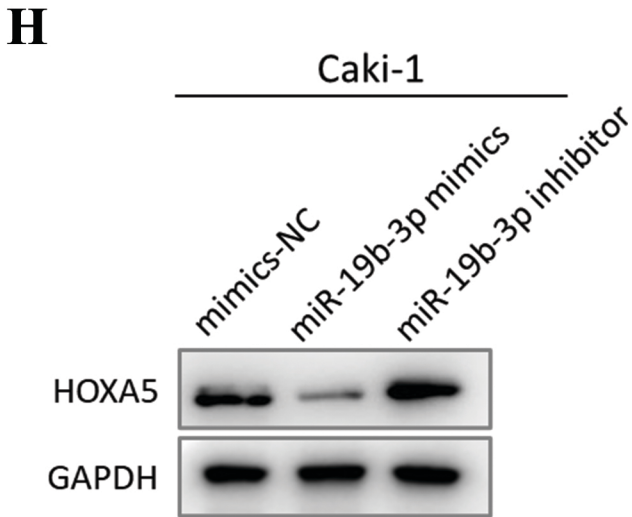

Supplement: Supplementary file 2 — Additional file 2 Fig. S2 The clinical properties of miR-19b-3p in RCC and its function in RCC cells. a. The pathways regulated by miR-19b-3p. Data was obtained from online database mirPath. Count indicated that the number of miRNA-related genes involved in the pathway. b. Expression analysis of miR-19b-3p in tumors and normal adjacent tissues in our patient cohort (total 102 patients). c. Survival analysis (overall survival) of miR-19b-3p in our patient cohort. d. Survival analysis (recurrent-free survival) of miR-19b-3p in our patient cohort. e. Expression analysis of miR-19b-3p in renal tumors from RCC patients with/without distant metastasis at the time of diagnosis (total 28 patients). f. qRT-PCR screening of 6 candidate mRNAs in 769P cells treated with mimics NC/miR-19b-3p mimics. Mimics NC group was used as normalization control. g. Immunoblotting of HOXA5 in 769P cells with treatment of miR-19b-3p mimics or miR-19b-3p inhibitor. GAPDH was used as internal control. h. Immunoblotting of HOXA5 in Caki-1 cells with treatment of miR-19b-3p mimics or miR-19b-3p inhibitor. GAPDH was used as internal control. Data are mean ± SD, n = 3. [file 12943_2022_1694_MOESM2_ESM.pdf]

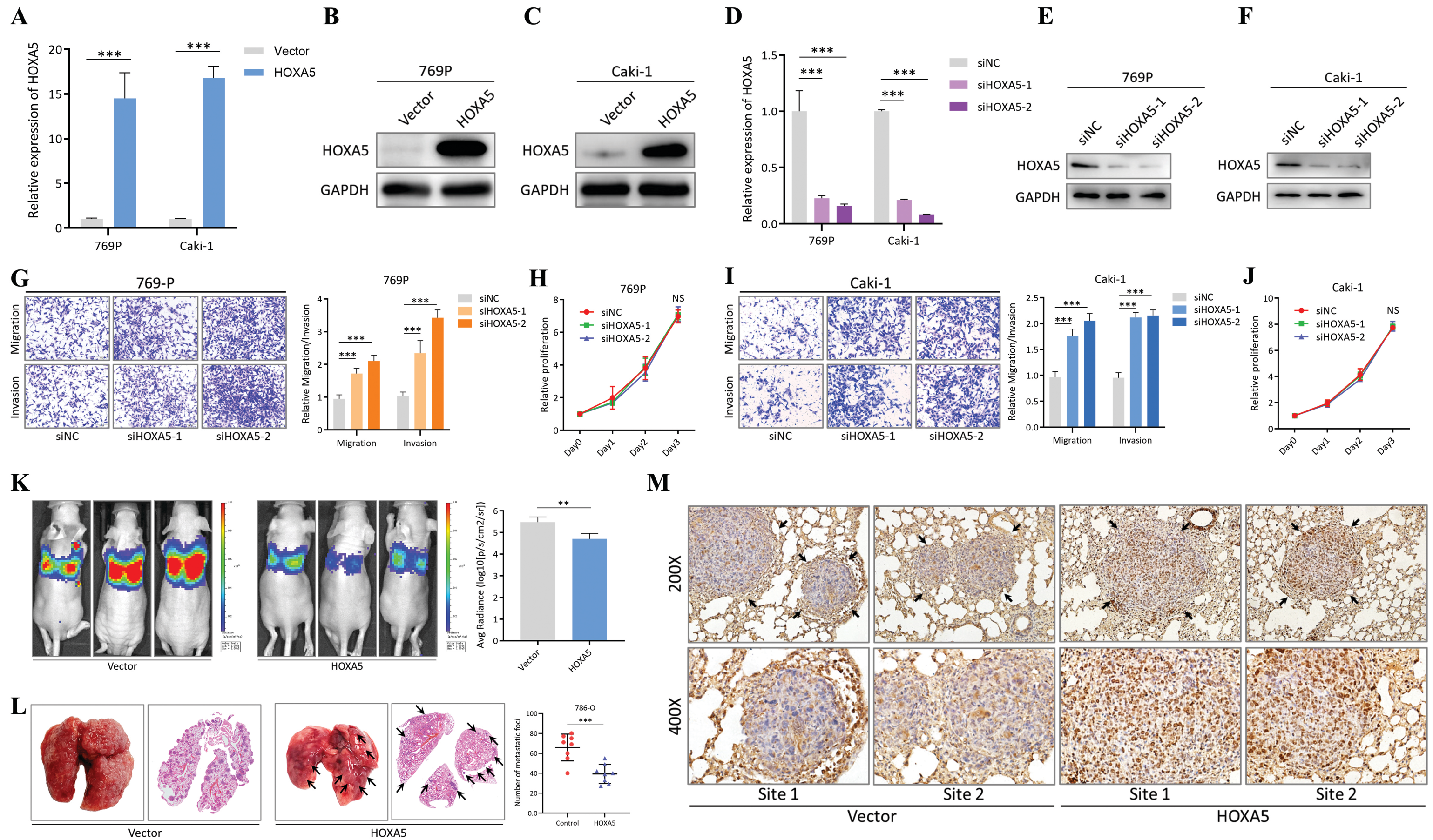

Supplement: Supplementary file 3 — Additional file 3 Fig. S3 HOXA5 inhibited the migration and invasion of RCC cells. a. qRT-PCR quantification of HOXA5 in 769P and Caki-1 cells transfected with control vector/HOXA5-overexpression. The control vector group was used as normalization. b. Immunoblotting confirmation of HOXA5 overexpression in 769P. GAPDH was used as normalization control. c. Immunoblotting confirmation of HOXA5 overexpression in Caki-1. GAPDH was used as normalization control. d. qRT-PCR quantification of HOXA5 in 769P/Caki-1 cells treated with control siNC/siHOXA5 siRNAs. The siNC group was used as normalization. e. Immunoblotting confirmation of HOXA5 knockdown in 769P. GAPDH was used as normalization control. f. Immunoblotting confirmation of HOXA5 knockdown in Caki-1. GAPDH was used as normalization control. g. Representative images (left) and quantification (right) data of Transwell migration/invasion assay of 769P cells treated with control siNC/siHOXA5 siRNAs. h. Proliferative activity of 769P cells treated with control siNC/siHOXA5 siRNAs measured by CCK8 assay. i. Representative images (left) and quantification (right) data of Transwell migration/invasion assay of Caki-1 cells treated with control siNC/siHOXA5 siRNAs. j. Proliferative activity of Caki-1 cells treated with control siNC/siHOXA5 siRNAs measured by CCK8 assay. k. Representative images (left) and in vivo luciferase activity quantification (right) data of mouse tail-vein lung metastasis model. 786-O cells transfected with vector/HOXA5-overexpression plasmid were used in the injection. Images were taken 8 weeks after the injection. l. Representative HE-stain images (left) and lung-metastasis foci quantification (right) data of 786-O mouse tail-vein lung metastasis model. m. Representative IHC images of HOXA5 in lung-metastasis foci from 786-O vector/HOXA5-overexpression group. Data are mean ± SD, n = 3. [file 12943_2022_1694_MOESM3_ESM.pdf]

**A**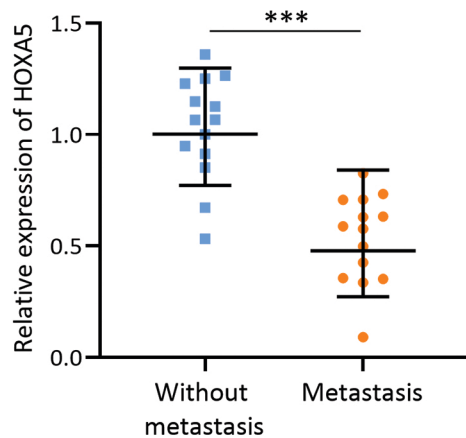**B**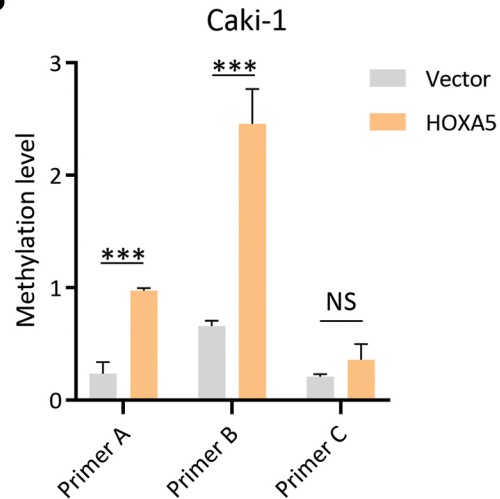**C**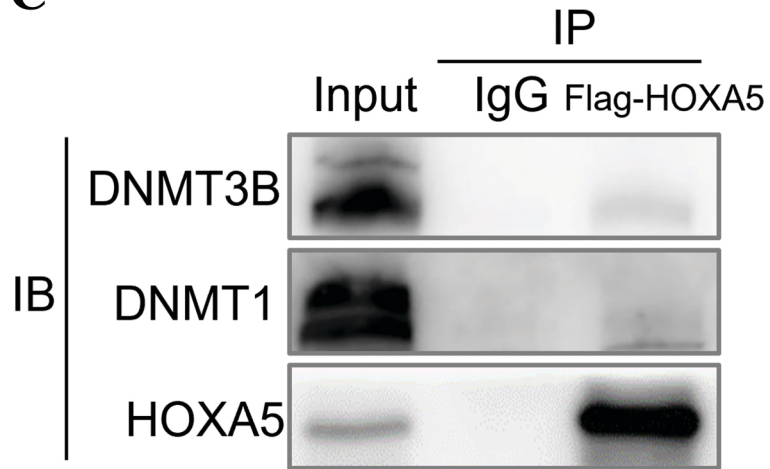

Supplement: Supplementary file 4 — Additional file 4 Fig. S4 HOXA5 recruited DNMT3A to increase the DNA methylation level in the promoter region of Slug. a. Expression analysis of HOXA5 in renal tumors from RCC patients with/without distant metastasis at the time of diagnosis (total 28 patients). b. MeDIP assay on Caki-1 cells with vector/HOXA5-overexpression conditions. Primer A, B and C were specific to different region of the Slug promoter. c. Immunoprecipitation using Flag antibody in Flag-HOXA5 overexpression cells. Non-specific IgG was used as negative control. Data are mean ± SD, n = 3. [file 12943_2022_1694_MOESM4_ESM.pdf]

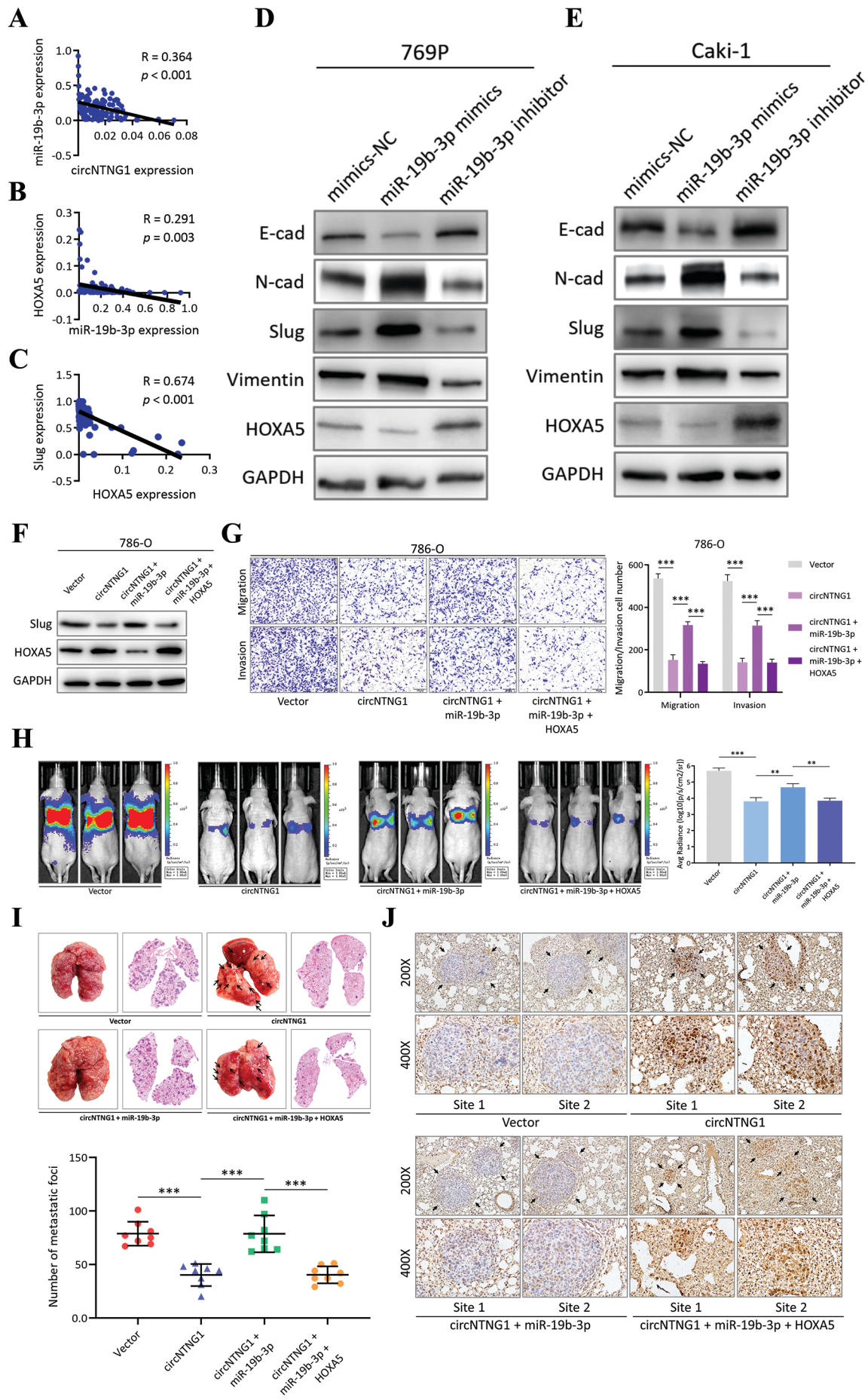

Supplement: Supplementary file 5 — Additional file 5 Fig. S5 The regulation of RCC by circNTNG1/miR-19b-3p/HOXA5 axis. a. Correlation analysis of circNTNG1 and miR-19b-3p in our own patient cohort (102 patients). b. Correlation analysis of miR-19b-3p and HOXA5 in our own patient cohort (102 patients). c. Correlation analysis of HOXA5 and Slug in our own patient cohort (102 patients). d. Immunoblotting of HOXA5, Slug and EMT markers with treatment of miR-19b-3p mimics or miR-19b-3p inhibitor in 769P cells. GAPDH was used as internal control. e. Immunoblotting of HOXA5, Slug and EMT markers with treatment of miR-19b-3p mimics or miR-19b-3p inhibitor in Caki-1 cells. GAPDH was used as positive control. f. Immunoblotting of HOXA5 and Slug in 786-O cells circNTNG1 overexpression salvage experiment. Cells were treated with circNTNG1 overexpression with/without miR-19b-3p mimics, and further salvaged with HOXA5 overexpression. GAPDH was used as positive control. g. Representative images (left) and quantification (right) data of Transwell migration/invasion assay of 786-O cells circNTNG1 overexpression salvage experiment. h. Representative images (left) and in vivo luciferase activity quantification (right) data of mouse tail-vein lung metastasis model. 786-O cells with vector, circNTNG1-overexpression, circNTNG1-overexpression+miR-19b-3p overexpression or circNTNG1-overexpression+miR-19b-3p overexpression+HOXA5 overexpression were used in the injection. Images were taken 8 weeks after the injection. i. Representative HE-stain images (left) and lung-metastasis foci quantification (right) data of 786-O mouse tail-vein lung metastasis model. j. Representative IHC images of HOXA5 in lung-metastasis foci from 786-O mouse tail-vein lung metastasis model. Data are mean ± SD, n = 3. [file 12943_2022_1694_MOESM5_ESM.pdf]
