# Supplementary material for: CircNTNG1 inhibits renal cell carcinoma progression via HOXA5-mediated epigenetic silencing of Slug
Source: Mol Cancer. 2022 Dec 19;21:224. doi: 10.1186/s12943-022-01694-7 (PMC9761964; doi:10.1186/s12943-022-01694-7)
Supplement: Supplementary file 6 — Additional file 6 Table S1 Primers and DNA/RNA sequence used in this study. [file 12943_2022_1694_MOESM6_ESM.docx]

**Additional file 6: Table S1**

| Primers and DNA/RNA sequence used in this study | |
| --- | --- |
| **Item** | **Sequence (5′-3′)** |
| **Primers** |  |
| GAPDH F | CTGGGCTACACTGAGCACC |
| GAPDH R | AAGTGGTCGTTGAGGGCAATG |
| HOXA5 F | AACTCATTTTGCGGTCGCTAT |
| HOXA5 R | TCCCTGAATTGCTCGCTCAC |
| E-cadherin F | CGAGAGCTACACGTTCACGG |
| E-cadherin R | GGGTGTCGAGGGAAAAATAGG |
| Slug F | CGAACTGGACACACATACAGTG |
| Slug R | CTGAGGATCTCTGGTTGTGGT |
| Primer 1 (-383 to -498 bp in HOXA5 ChIP-qPCR) F | CCGCTTCCCCCTTCCTTTTT |
| Primer 1 (-383 to -498 bp in HOXA5 ChIP-qPCR) R | GGCCAGCCTCTGGTGTTAAT |
| Primer 2 (-699 to -808 bp in HOXA5 ChIP-qPCR) F | GTGAGAGAATGTCCGGTGGT |
| Primer 2 (-699 to -808 bp in HOXA5 ChIP-qPCR) R | AAAGGCAGGCTGATCGGAAG |
| Primer 3 (-1323 to -1418 bp in HOXA5 ChIP-qPCR) F | GGAAATCTGTGAGTGCCCCA |
| Primer 3 (-1323 to -1418 bp in HOXA5 ChIP-qPCR) R | AGTGCATTGTCAGGTTGCAT |
| Primer A (-249 to -436 bp in HOXA5 MeDIP-qPCR) F | AGCACCACATAAAAGCAGGG |
| Primer A (-249 to -436 bp in HOXA5 MeDIP-qPCR) R | TGGAACCACCGGACATTCTC |
| Primer B (-625 to -708 bp in HOXA5 MeDIP-qPCR) F | GCCTGCCTTTAGAGGGCTAC |
| Primer B (-625 to -708 bp in HOXA5 MeDIP-qPCR) R | GGCCGCGTGCAAATTAAGTA |
| Primer C (-785 to -937 bp in HOXA5 MeDIP-qPCR) F | CCTCTCCAGATGCCACTTCC |
| Primer C (-785 to -937 bp in HOXA5 MeDIP-qPCR) R | CTCTCTGGGAGCTAGGAGGG |
| circNTNG1 F | GGTGCGAGGAAGGGCAAT |
| circNTNG1 R | ATAGGGCTGCCATGTTCGTC |
| NTNG1 F | GGATCTGCTTTGAGGTCCCA |
| NTNG1 R | AAAGGGTAGGGCTGCATCAC |
| miRNA RT primer | CAGTGCAGGGTCCGAGGTCAGAGCCACCTGGGCAATTTTTTTTTTTVN |
| miR-19b-3p F | CAGTGCAGGGTCCGAGGT |
| miR-19b-3p R | AAGCGACCTGTGCAAATCCATG |
| NR3C2 F | GAAAGACGGTGGGGTCAAGTT |
| NR3C2 R | ACCGGAAACACAGCTTACGTT |
| PPARA F | TTCGCAATCCATCGGCGAG |
| PPARA R | CCACAGGATAAGTCACCGAGG |
| SOX6 F | GGATGCAATGACCCAGGATTT |
| SOX6 R | TGAATGGTACTGACAAGTGTTGG |
| SMARCA2 F | AGGGGATTGTAGAAGACATCCA |
| SMARCA2R | TTGGCTGTGTTGATCCATTGG |
| SATB1 F | GATCATTTGAACGAGGCAACTCA |
| SATB1 R | TGGACCCTTCGGATCACTCA |
| **siRNAs** |  |
| siHOXA5-1 | CGGCTACAATGGCATGGAT |
| siHOXA5-2 | GCAGAAGGAGGATTGAAAT |
| siDNMT3A-1 | CCACCAAAGCAGGCGATGA |
| siDNMT3A-2 | CCACGACAGCGATGAGAGT |
| **miRNA mimics and inhibitors** |  |
| miR-19b-3p mimics | UGUGCAAAUCCAUGCAAAACUGA |
| miR-19b-3p inhibitor | UCAGUUUUGCAUGGAUUUGCACA |
| **Luciferase reporter assay** |  |
| HOXA5-WT 3’UTR | GCCUUUAUAGGACCCUUUGCACG |
| HOXA5-Mut 3’UTR | GCCUUUAUAGGACCCGGCTUCTG |
| circNTNG1 | GGCAATCCCTACATGTGCAATAATGAGTGTGATGCGAGTACCCCTGAGCTGGCACACCCCCCTGAGCTGATGTTTGATTTTGAAGGAAGACATCCCTCCACATTTTGGCAGTCTGCCACTTGGAAGGAGTATCCCAAGCCTCTCCAGGTTAACATCACTCTGTCTTGGAGCAAAACCATTGAGCTAACAGACAACATAGTTATTACCTTTGAATCTGGGCGTCCAGACCAAATGATCCTGGAGAAGTCTCTCGATTATGGACGAACATGGCAGCCCTATCAGTATTATGCCACAGACTGCTTAGATGCTTTTCACATGGATCCTAAATCCGTGAAGGATTTATCACAGCATACGGTCTTAGAAATCATTTGCACAGAAGAGTACTCAACAGGGTATACAACAAATAGCAAAATAATCCACTTTGAAATCAAAGACAGGTTCGCGTTTTTTGCTGGACCTCGCCTACGCAATATGGCTTCCCTCTACGGACAGCTGGATACAACCAAGAAACTCAGAGATTTCTTTACAGTCACAGACCTGAGGATAAGGCTGTTAAGACCAGCCGTTGGGGAAATATTTGTAGATGAGCTACACTTGGCACGCTACTTTTACGCGATCTCAGACATAAAGGTGCGAGGAAG |
| circNTNG1-Mut | CCGTTAGGGATGTACACGTTATTACTCACACTACGCTCATGGGGACTCGACCGTGTGGGGGGACTCGACTACAAACTAAAACTTCCTTCTGTAGGGAGGTGTAAAACCGTCAGACGGTGAACCTTCCTCATAGGGTTCGGAGAGGTCCAATTGTAGTGAGACAGAACCTCGTTTTGGTAACTCGATTGTCTGTTGTATCAATAATGGAAACTTAGACCCGCAGGTCTGGTTTACTAGGACCTCTTCAGAGAGCTAATACCTGCTTGTACCGTCGGGATAGTCATAATACGGTGTCTGACGAATCTACGAAAAGTGTACCTAGGATTTAGGCACTTCCTAAATAGTGTCGTATGCCAGAATCTTTAGTAAACGTGTCTTCTCATGAGTTGTCCCATATGTTGTTTATCGTTTTATTAGGTGAAACTTTAGTTTCTGTCCAAGCGCAAAAAACGACCTGGAGCGGATGCGTTATACCGAAGGGAGATGCCTGTCGACCTATGTTGGTTCTTTGAGTCTCTAAAGAAATGTCAGTGTCTGGACTCCTATTCCGACAATTCTGGTCGGCAACCCCTTTATAAACATCTACTCGATGTGAACCGTGCGATGAAAATGCGCTAGAGTCTGTATTTCCACGCTCCTTC |
