# Supplementary material for: CircNTNG1 inhibits renal cell carcinoma progression via HOXA5-mediated epigenetic silencing of Slug
Source: Mol Cancer. 2022 Dec 19;21:224. doi: 10.1186/s12943-022-01694-7 (PMC9761964; doi:10.1186/s12943-022-01694-7)
Supplement: Supplementary file 7 — Additional file 7 Table S2 Clinical information of patient samples in the circRNA-seq. [file 12943_2022_1694_MOESM7_ESM.docx]

**Additional file 7: Table S2**

| Clinical information of patient samples in the circRNA-seq | | | | | | | |  |  |  |  |  |
| --- | --- | --- | --- | --- | --- | --- | --- | --- | --- | --- | --- | --- |
| **Sample number** | **Age** | **Gender** | **Tumor location** | **Tumor size (CT scan)** | **Perirenal invasion (CT scan)** | **Clinical TNM** | **Type of surgery** | **Diagnosis** | **Tumor size (pathology)** | **Perirenal invasion (pathology)** | **Fuhrman grade** | **Pathologic TNM** |
| 30697999 | 80 | Female | Left | 5.4cm | No | cT_1b_N_0_M_0_ | Laparoscopic radical nephrectomy | ccRCC | 4.5cm | No | Grade 2 | pT_1b_N_0_M_0_ |
| 30698963 | 50 | Male | Left | 3.4cm | No | cT_1a_N_0_M_0_ | Laparoscopic partial nephrectomy | ccRCC | 4.0cm | No | Grade 2 | pT_1a_N_0_M_0_ |
| 30694710 | 47 | Male | Right | 5.5cm | Yes | cT_3a_N_0_M_0_ | Radical nephrectomy | ccRCC | 6.0cm | No | Grade 3 | pT_1b_N_0_M_0_ |
| 30695021 | 60 | Male | Right | 9.9cm | Yes | cT_3a_N_0_M_0_ | Laparoscopic radical nephrectomy | ccRCC | 8.0cm | No | Grade 2 | pT_2a_N_0_M_0_ |
| 30699785 | 51 | Male | Left | 3.7cm | Yes | cT_3a_N_0_M_0_ | Radical nephrectomy | ccRCC | 4.0cm | Yes | Grade 2 | pT_3a_N_0_M_0_ |
