# Supplementary material for: CircNTNG1 inhibits renal cell carcinoma progression via HOXA5-mediated epigenetic silencing of Slug
Source: Mol Cancer. 2022 Dec 19;21:224. doi: 10.1186/s12943-022-01694-7 (PMC9761964; doi:10.1186/s12943-022-01694-7)
Supplement: Supplementary file 8 — Additional file 8 Table S3 Frequently Deleted Chromosomes in RCC. [file 12943_2022_1694_MOESM8_ESM.docx]

**Additional file 8: Table S3**

| Frequently Deleted Chromosomes in RCC | |
| --- | --- |
| **Reference** | **Frequently Deleted Chromosomes in RCC** |
| [15] | 3p、8 |
| [16] | 3p |
| [17] | 1, 9, 22 |
