# Supplementary material for: CircNTNG1 inhibits renal cell carcinoma progression via HOXA5-mediated epigenetic silencing of Slug
Source: Mol Cancer. 2022 Dec 19;21:224. doi: 10.1186/s12943-022-01694-7 (PMC9761964; doi:10.1186/s12943-022-01694-7)
Supplement: Supplementary file 9 — Additional file 9 Table S4 Significant circRNAs after filtration in our own dataset. [file 12943_2022_1694_MOESM9_ESM.docx]

**Additional file 9: Table S4**

| Significant circRNAs after filtration in our own dataset | | | | | | | |
| --- | --- | --- | --- | --- | --- | --- | --- |
| **circRNA** | **circbase ID** | **Chrom** | **txStart** | **txEnd** | **Gene symbol** | **log2FC** | **P value** |
| hsa_circ:chr1:107866904-107867544 | hsa_circ_0002286 | chr1 | 107866904 | 107867544 | NTNG1 | -6.524162865 | 4.61E-13 |
| hsa_circ:chr8:102570647-102571040 | hsa_circ_0085173 | chr8 | 102570647 | 102571040 | GRHL2 | -5.861659071 | 8.14E-08 |
| hsa_circ:chr3:150834125-150845771 | hsa_circ_0067735 | chr3 | 150834125 | 150845771 | MED12L | -4.813882379 | 1.31E-05 |
| hsa_circ:chr3:77147165-77147491 | hsa_circ_0066556 | chr3 | 77147165 | 77147491 | ROBO2 | -4.508426299 | 0.001520968 |
| hsa_circ:chr9:118969735-119033695 | hsa_circ_0008792 | chr9 | 118969735 | 119033695 | PAPPA | -4.348996583 | 0.004957841 |
| hsa_circ:chr9:34234214-34242106 | hsa_circ_0086743 | chr9 | 34234214 | 34242106 | UBAP1 | -3.884278525 | 0.042379935 |
| hsa_circ:chr3:44970798-45000952 | hsa_circ_0065045 | chr3 | 44970798 | 45000952 | ZDHHC3 | -3.756530801 | 0.035491137 |
| hsa_circ:chr8:116599228-116635985 | hsa_circ_0085362 | chr8 | 116599228 | 116635985 | TRPS1 | -3.741348634 | 5.37E-04 |
| hsa_circ:chr3:17051166-17056403 | hsa_circ_0001274 | chr3 | 17051166 | 17056403 | PLCL2 | -3.64070064 | 6.44E-05 |
| hsa_circ:chr9:133364720-133370410 | hsa_circ_0089107 | chr9 | 133364720 | 133370410 | ASS1 | -3.475257728 | 0.010523449 |
| hsa_circ:chr8:10065343-10177499 | hsa_circ_0002546 | chr8 | 10065343 | 10177499 | MSRA | -3.467539508 | 0.006715723 |
| hsa_circ:chr3:113497588-113505230 | hsa_circ_0066826 | chr3 | 113497588 | 113505230 | ATP6V1A | -3.218425621 | 0.004127815 |
| hsa_circ:chr3:14197835-14201330 | hsa_circ_0064423 | chr3 | 14197835 | 14201330 | XPC | -3.184436995 | 0.009689278 |
| hsa_circ:chr1:100960374-100964818 | hsa_circ_0013330 | chr1 | 100960374 | 100964818 | CDC14A | -3.149805026 | 0.001948735 |
| hsa_circ:chr3:107429299-107474525 | hsa_circ_0066735 | chr3 | 107429299 | 107474525 | BBX | -3.147375992 | 0.001061323 |
| hsa_circ:chr1:11184555-11193254 | hsa_circ_0006576 | chr1 | 11184555 | 11193254 | MTOR | -3.135841249 | 0.006689688 |
| hsa_circ:chr8:17532695-17581342 | hsa_circ_0083441 | chr8 | 17532695 | 17581342 | MTUS1 | -2.949538701 | 1.68E-04 |
| hsa_circ:chr8:121013764-121021372 | hsa_circ_0085412 | chr8 | 121013764 | 121021372 | DEPTOR | -2.945663914 | 0.016160178 |
| hsa_circ:chr9:128246722-128268696 | hsa_circ_0002303 | chr9 | 128246722 | 128268696 | MAPKAP1 | -2.911246324 | 0.012263564 |
| hsa_circ:chr9:16552528-16583083 | hsa_circ_0086416 | chr9 | 16552528 | 16583083 | BNC2 | -2.909743075 | 0.00135861 |
| hsa_circ:chr8:116616100-116635985 | hsa_circ_0085363 | chr8 | 116616100 | 116635985 | TRPS1 | -2.89201883 | 0.049368511 |
| hsa_circ:chr3:197557641-197593090 | hsa_circ_0068738 | chr3 | 197557641 | 197593090 | ENSG00000234136.1,LRCH3 | -2.735777001 | 0.001327885 |
| hsa_circ:chr3:50142982-50145002 | hsa_circ_0065805 | chr3 | 50142982 | 50145002 | RBM5 | -2.628750756 | 0.010053454 |
| hsa_circ:chr3:65464267-65479306 | hsa_circ_0066459 | chr3 | 65464267 | 65479306 | MAGI1 | -2.520882358 | 0.015379763 |
| hsa_circ:chr3:183368084-183390272 | hsa_circ_0006667 | chr3 | 183368084 | 183390272 | KLHL24 | -2.50694227 | 8.26E-04 |
| hsa_circ:chr3:33633887-33686395 | hsa_circ_0064772 | chr3 | 33633887 | 33686395 | CLASP2 | -2.477193488 | 0.018345739 |
| hsa_circ:chr9:128230251-128268696 | hsa_circ_0003984 | chr9 | 128230251 | 128268696 | MAPKAP1 | -2.38596512 | 0.00196471 |
| hsa_circ:chr3:126340585-126366139 | hsa_circ_0008181 | chr3 | 126340585 | 126366139 | TXNRD3 | -2.351980468 | 9.17E-04 |
| hsa_circ:chr9:119093523-119097353 | hsa_circ_0088239 | chr9 | 119093523 | 119097353 | PAPPA | -2.332242567 | 0.011940266 |
| hsa_circ:chr8:135612679-135622898 | hsa_circ_0007209 | chr8 | 135612679 | 135622898 | ZFAT,ZFAT-AS1 | -2.305959707 | 9.43E-05 |
| hsa_circ:chr8:107691438-107705105 | hsa_circ_0085311 | chr8 | 107691438 | 107705105 | OXR1 | -2.271281612 | 0.002786535 |
| hsa_circ:chr3:127965679-127983624 | hsa_circ_0067209 | chr3 | 127965679 | 127983624 | EEFSEC | -2.264241023 | 0.018849852 |
| hsa_circ:chr8:18622959-18662408 | hsa_circ_0002111 | chr8 | 18622959 | 18662408 | PSD3 | -2.215388678 | 7.95E-06 |
| hsa_circ:chr3:47680215-47719801 | hsa_circ_0065256 | chr3 | 47680215 | 47719801 | SMARCC1 | -2.177629672 | 0.008354516 |
| hsa_circ:chr3:67546222-67579610 | hsa_circ_0005657 | chr3 | 67546222 | 67579610 | SUCLG2 | -2.167721728 | 1.84E-04 |
| hsa_circ:chr9:115013209-115060196 | hsa_circ_0003458 | chr9 | 115013209 | 115060196 | PTBP3 | -2.163488197 | 4.39E-04 |
| hsa_circ:chr3:65415195-65416556 | hsa_circ_0066452 | chr3 | 65415195 | 65416556 | MAGI1 | -2.160432878 | 3.28E-06 |
| hsa_circ:chr8:136554897-136594316 | hsa_circ_0006059 | chr8 | 136554897 | 136594316 | KHDRBS3 | -2.12818524 | 0.048002669 |
| hsa_circ:chr1:12335882-12338095 | hsa_circ_0009964 | chr1 | 12335882 | 12338095 | VPS13D | -2.127804594 | 2.31E-05 |
| hsa_circ:chr8:15508206-15531345 | hsa_circ_0006410 | chr8 | 15508206 | 15531345 | TUSC3 | -2.105147499 | 0.006463802 |
| hsa_circ:chr3:183361268-183390272 | hsa_circ_0001368 | chr3 | 183361268 | 183390272 | KLHL24 | -2.073205203 | 1.37E-04 |
| hsa_circ:chr3:47703824-47727660 | hsa_circ_0065266 | chr3 | 47703824 | 47727660 | SMARCC1 | -2.055339767 | 0.033591901 |
| hsa_circ:chr3:65415195-65428524 | hsa_circ_0003540 | chr3 | 65415195 | 65428524 | MAGI1 | -2.032572065 | 3.13E-04 |
| hsa_circ:chr3:183361268-183369064 | hsa_circ_0001367 | chr3 | 183361268 | 183369064 | KLHL24 | -2.026111686 | 8.66E-07 |
| hsa_circ:chr8:17601113-17613470 | hsa_circ_0083444 | chr8 | 17601113 | 17613470 | MTUS1 | -2.021828071 | 0.043709018 |
| hsa_circ:chr3:18419662-18462483 | hsa_circ_0064555 | chr3 | 18419662 | 18462483 | SATB1 | -1.947858074 | 0.00524415 |
| hsa_circ:chr9:111843119-111849622 | hsa_circ_0004504 | chr9 | 111843119 | 111849622 | TMEM245 | -1.921698165 | 0.009859478 |
| hsa_circ:chr8:133848785-133856563 | hsa_circ_0085644 | chr8 | 133848785 | 133856563 | PHF20L1 | -1.891366717 | 0.034018312 |
| hsa_circ:chr1:100889778-100908552 | hsa_circ_0000097 | chr1 | 100889778 | 100908552 | CDC14A | -1.880423045 | 0.02071597 |
| hsa_circ:chr3:43640024-43647355 | hsa_circ_0003533 | chr3 | 43640024 | 43647355 | ANO10 | -1.871896479 | 0.048933969 |
| hsa_circ:chr3:43591212-43607219 | hsa_circ_0009084 | chr3 | 43591212 | 43607219 | ANO10 | -1.866619925 | 0.005233542 |
| hsa_circ:chr9:20819795-20885229 | hsa_circ_0086536 | chr9 | 20819795 | 20885229 | FOCAD | -1.85427265 | 0.044716774 |
| hsa_circ:chr8:17532695-17573410 | hsa_circ_0083440 | chr8 | 17532695 | 17573410 | MTUS1 | -1.848140166 | 0.034185284 |
| hsa_circ:chr3:47098311-47108608 | hsa_circ_0002569 | chr3 | 47098311 | 47108608 | SETD2 | -1.837950832 | 0.012162001 |
| hsa_circ:chr8:28570974-28575724 | hsa_circ_0003885 | chr8 | 28570974 | 28575724 | EXTL3 | -1.791796749 | 0.02757604 |
| hsa_circ:chr8:17570723-17581342 | hsa_circ_0083443 | chr8 | 17570723 | 17581342 | MTUS1 | -1.788259904 | 1.36E-06 |
| hsa_circ:chr1:118003111-118045592 | hsa_circ_0002059 | chr1 | 118003111 | 118045592 | MAN1A2 | -1.752866403 | 8.87E-04 |
| hsa_circ:chr3:47651556-47663831 | hsa_circ_0008997 | chr3 | 47651556 | 47663831 | SMARCC1 | -1.686204615 | 0.019683691 |
| hsa_circ:chr9:136302869-136303486 | hsa_circ_0089372 | chr9 | 136302869 | 136303486 | ADAMTS13 | -1.683898889 | 0.014124323 |
| hsa_circ:chr3:113505079-113508687 | hsa_circ_0002640 | chr3 | 113505079 | 113508687 | ATP6V1A | -1.67707914 | 0.044820252 |
| hsa_circ:chr9:16727795-16738483 | hsa_circ_0008732 | chr9 | 16727795 | 16738483 | BNC2 | -1.675513735 | 2.17E-05 |
| hsa_circ:chr9:126519982-126641300 | hsa_circ_0002544 | chr9 | 126519982 | 126641300 | DENND1A | -1.665270265 | 1.21E-08 |
| hsa_circ:chr3:33661095-33686395 | hsa_circ_0064784 | chr3 | 33661095 | 33686395 | CLASP2 | -1.654674192 | 0.043716699 |
| hsa_circ:chr3:16327849-16345099 | hsa_circ_0001273 | chr3 | 16327849 | 16345099 | OXNAD1 | -1.647404274 | 1.99E-04 |
| hsa_circ:chr3:47079156-47108608 | hsa_circ_0004692 | chr3 | 47079156 | 47108608 | SETD2 | -1.631820836 | 0.001701143 |
| hsa_circ:chr3:47770515-47814426 | hsa_circ_0005435 | chr3 | 47770515 | 47814426 | SMARCC1 | -1.628728995 | 0.001286574 |
| hsa_circ:chr8:19683937-19694671 | hsa_circ_0083500 | chr8 | 19683937 | 19694671 | INTS10 | -1.593812838 | 0.0179438 |
| hsa_circ:chr9:115024715-115060196 | hsa_circ_0008192 | chr9 | 115024715 | 115060196 | PTBP3 | -1.557677262 | 0.001279958 |
| hsa_circ:chr9:4117768-4118881 | hsa_circ_0006370 | chr9 | 4117768 | 4118881 | GLIS3 | -1.526830407 | 0.00428309 |
| hsa_circ:chr9:33935837-33941860 | hsa_circ_0008344 | chr9 | 33935837 | 33941860 | UBAP2 | -1.519738334 | 0.040676441 |
| hsa_circ:chr3:71064700-71102924 | hsa_circ_0001320 | chr3 | 71064700 | 71102924 | FOXP1 | -1.519394406 | 0.015660698 |
| hsa_circ:chr3:12421203-12422990 | hsa_circ_0064338 | chr3 | 12421203 | 12422990 | PPARG | -1.510338883 | 0.012711587 |
| hsa_circ:chr3:44986660-45000952 | hsa_circ_0065052 | chr3 | 44986660 | 45000952 | ZDHHC3 | -1.493750438 | 0.022292389 |
| hsa_circ:chr3:61975299-61989171 | hsa_circ_0066406 | chr3 | 61975299 | 61989171 | PTPRG | -1.489022233 | 0.00117785 |
| hsa_circ:chr3:185183535-185184751 | hsa_circ_0068390 | chr3 | 185183535 | 185184751 | MAP3K13 | -1.415031729 | 0.043043934 |
| hsa_circ:chr3:37315027-37323763 | hsa_circ_0008654 | chr3 | 37315027 | 37323763 | GOLGA4 | -1.399632191 | 0.020956671 |
| hsa_circ:chr9:111812563-111835718 | hsa_circ_0004010 | chr9 | 111812563 | 111835718 | TMEM245 | -1.39766654 | 0.006753347 |
| hsa_circ:chr3:47777524-47814426 | hsa_circ_0005367 | chr3 | 47777524 | 47814426 | SMARCC1 | -1.365585064 | 0.018641409 |
| hsa_circ:chr9:35295693-35313986 | hsa_circ_0008518 | chr9 | 35295693 | 35313986 | UNC13B | -1.316859278 | 0.001369021 |
| hsa_circ:chr9:35546427-35548532 | hsa_circ_0002702 | chr9 | 35546427 | 35548532 | RUSC2 | -1.313750505 | 0.0024553 |
| hsa_circ:chr3:31617888-31641951 | hsa_circ_0003338 | chr3 | 31617888 | 31641951 | STT3B | -1.299185753 | 0.047935981 |
| hsa_circ:chr3:31917925-31921322 | hsa_circ_0008549 | chr3 | 31917925 | 31921322 | OSBPL10 | -1.266073601 | 0.012334622 |
| hsa_circ:chr9:128419930-128434922 | hsa_circ_0001890 | chr9 | 128419930 | 128434922 | MAPKAP1 | -1.257659805 | 0.021213899 |
| hsa_circ:chr1:104108057-104118162 | hsa_circ_0000099 | chr1 | 104108057 | 104118162 | AMY2B | -1.253125593 | 0.017115093 |
| hsa_circ:chr3:110830877-110845182 | hsa_circ_0066776 | chr3 | 110830877 | 110845182 | NECTIN3 | -1.237748887 | 0.045915978 |
| hsa_circ:chr3:56694759-56707753 | hsa_circ_0001315 | chr3 | 56694759 | 56707753 | FAM208A | -1.230675705 | 0.00343715 |
| hsa_circ:chr8:28821328-28837673 | hsa_circ_0001995 | chr8 | 28821328 | 28837673 | HMBOX1 | -1.187328546 | 0.011329328 |
| hsa_circ:chr8:37971710-37978667 | hsa_circ_0006302 | chr8 | 37971710 | 37978667 | ASH2L | -1.154145643 | 0.020939377 |
| hsa_circ:chr8:106431372-106456609 | hsa_circ_0003380 | chr8 | 106431372 | 106456609 | ZFPM2 | -1.153303565 | 0.012817619 |
| hsa_circ:chr9:114332371-114348445 | hsa_circ_0008043 | chr9 | 114332371 | 114348445 | PTGR1 | -1.109583128 | 0.01258627 |
| hsa_circ:chr9:115030329-115060196 | hsa_circ_0003500 | chr9 | 115030329 | 115060196 | PTBP3 | -1.108350061 | 0.015051829 |
| hsa_circ:chr8:22332467-22355625 | hsa_circ_0006320 | chr8 | 22332467 | 22355625 | PPP3CC | -1.087787388 | 0.020695337 |
| hsa_circ:chr1:115005726-115006178 | hsa_circ_0013637 | chr1 | 115005726 | 115006178 | TRIM33 | -1.074082788 | 0.043912871 |
| hsa_circ:chr3:149563798-149629870 | hsa_circ_0003502 | chr3 | 149563798 | 149629870 | RNF13 | -1.059119649 | 0.043910262 |
| hsa_circ:chr3:67546222-67559327 | hsa_circ_0004276 | chr3 | 67546222 | 67559327 | SUCLG2 | -1.014202493 | 2.20E-04 |
| hsa_circ:chr3:71090479-71102924 | hsa_circ_0008234 | chr3 | 71090479 | 71102924 | FOXP1 | -1.00518782 | 0.031312778 |
| hsa_circ:chr8:42317414-42323435 | hsa_circ_0008015 | chr8 | 42317414 | 42323435 | SLC20A2 | -1.003964426 | 0.024673279 |
