# Supplementary material for: CircNTNG1 inhibits renal cell carcinoma progression via HOXA5-mediated epigenetic silencing of Slug
Source: Mol Cancer. 2022 Dec 19;21:224. doi: 10.1186/s12943-022-01694-7 (PMC9761964; doi:10.1186/s12943-022-01694-7)
Supplement: Supplementary file 10 — Additional file 10 Table S5 Significant circRNAs after filtration in two public datasets (GSE137836 and GSE100186). [file 12943_2022_1694_MOESM10_ESM.docx]

**Additional file 10: Table S5**

| Significant circRNAs after filtration in two public datasets (GSE137836 and GSE100186) | | | | | | | | | | |
| --- | --- | --- | --- | --- | --- | --- | --- | --- | --- | --- |
| **circRNA** | **circbase ID** | **Chrom** | **txStart** | **txEnd** | **Gene symbol** | **P value in GSE137836** | **log2FC in GSE137836** | **P value in GSE100186** | **log2FC in GSE100186** | **Average log2FC** |
| ASCRP3008571 | hsa_circ_0002286 | chr1 | 107866903 | 107867544 | NTNG1 | 0.0220849 | -2.724876 | 0.0097 | -3.5233103 | -3.12409315 |
| ASCRP3009671 | hsa_circ_0006226 | chr3 | 61607643 | 61609229 | PTPRG | 0.00147 | -2.621195 | 0.0141 | -2.2351749 | -2.42818495 |
| ASCRP3003109 | hsa_circ_0002453 | chr3 | 8977554 | 8990254 | RAD18 | 0.030204 | -1.819012 | 0.0143 | -2.4489439 | -2.13397795 |
| ASCRP3001546 | hsa_circ_0000026 | chr1 | 21377358 | 21437876 | EIF4G3 | 0.0485838 | -1.321204 | 0.024 | -2.8071885 | -2.06419625 |
| ASCRP3005857 | hsa_circ_0088479 | chr9 | 126554865 | 126641300 | DENND1A | 0.0137211 | -2.369328 | 0.0151 | -1.6300738 | -1.9997009 |
| ASCRP3012426 | hsa_circ_0005927 | chr8 | 42259305 | 42260979 | VDAC3 | 0.0305218 | -1.610065 | 0.0269 | -2.1822661 | -1.89616555 |
| ASCRP3011120 | hsa_circ_0000002 | chr1 | 1158623 | 1159348 | SDF4 | 0.029224 | -1.684928 | 0.0264 | -2.0033421 | -1.84413505 |
| ASCRP3002246 | hsa_circ_0002544 | chr9 | 126519981 | 126641300 | DENND1A | 0.0268353 | -2.253342 | 0.0357 | -1.3674347 | -1.81038835 |
| ASCRP3001545 | hsa_circ_0005075 | chr1 | 21377358 | 21415706 | EIF4G3 | 0.0451695 | -1.11134 | 0.025 | -2.4571821 | -1.78426105 |
| ASCRP3004961 | hsa_circ_0088478 | chr9 | 126531792 | 126641300 | DENND1A | 0.036305 | -2.123141 | 0.0465 | -1.3772603 | -1.75020065 |
| ASCRP3009299 | hsa_circ_0008508 | chr9 | 20907148 | 20929595 | FOCAD | 0.0161897 | -1.360618 | 0.0198 | -2.0045866 | -1.6826023 |
| ASCRP3001224 | hsa_circ_0000097 | chr1 | 100889777 | 100908552 | CDC14A | 0.0411479 | -1.899379 | 0.0296 | -1.2799023 | -1.58964065 |
| ASCRP3006626 | hsa_circ_0011474 | chr1 | 33760537 | 33766320 | ZNF362 | 0.0302523 | -1.151233 | 0.00771 | -1.9275119 | -1.53937245 |
| ASCRP3004669 | hsa_circ_0088467 | chr9 | 126392654 | 126641300 | DENND1A | 0.0222963 | -1.849806 | 0.0407 | -1.0386256 | -1.4442158 |
| ASCRP3006044 | hsa_circ_0009244 | chr1 | 1158623 | 1164326 | SDF4 | 0.0436219 | -1.175591 | 0.00131 | -1.6097336 | -1.3926623 |
| ASCRP3001609 | hsa_circ_0004390 | chr1 | 85331067 | 85331821 | LPAR3 | 0.035682 | -1.103685 | 0.00129 | -1.4319412 | -1.2678131 |
