# Supplementary material for: CircNTNG1 inhibits renal cell carcinoma progression via HOXA5-mediated epigenetic silencing of Slug
Source: Mol Cancer. 2022 Dec 19;21:224. doi: 10.1186/s12943-022-01694-7 (PMC9761964; doi:10.1186/s12943-022-01694-7)
Supplement: Supplementary file 11 — Additional file 11 Supplemental materials and methods Supplemental materials and methods. [file 12943_2022_1694_MOESM11_ESM.docx]

**Supplemental materials and methods**

**Cell lines and cell culture**

Four human RCC cell lines (786-O, 769P, Caki-1and A498) plus one normal human kidney cell line 293 were obtained from the Chinese Academy of Sciences. Roswell Park Memorial Institute (RPMI) 1640 (Gibco, USA) with 10% fetal bovine serum (FBS; PAN-Seratech, Germany) was used to culture 786-O and 769P. McCoy (Gibco) with 10% FBS (PAN-Seratech) was used to culture Caki-1 and A498. Dulbecco’s Modified Eagle Medium (Gibco) with 10% FBS (PAN-Seratech) was used to culture 293. All cells were incubated at 37°C in 5% CO_2_. Mycoplasma infection was routinely monitored for using a kit (Beyotime, China) during cell culture.

**Plasmid and small interfering RNA (siRNA) transfection**

The HOXA5, Flag-HOXA5, miR-19b-3p and circNTNG1 overexpression plasmids were synthesized by Genecreate (China). Empty vector was used as the negative control. The HOXA5 and DNMT3A siRNAs as well as the miR-19b-3p mimics and inhibitor were synthesized by RiboBio (China). The transfection was performed using Lipofectamine 3000 (Invitrogen, USA). Stable cell lines were established by using lentivirus preparations infection and puromycin (Biosharp, China) selection. Lentivirus preparations were produced in HEK-293T cells with target plasmid and packaging vectors.

**RNA extraction, cDNA synthesis, and quantitative real-time (qRT)-PCR**

Cells were lysed and total RNA was extracted with TRIzol (Invitrogen) according to the manufacturer’s protocol. Thereafter, the first strand of cDNA was synthesized using an iScript cDNA Synthesis Kit (Bio-Rad, USA). The stem-loop method was utilized to synthesize the first strand of cDNA for microRNA. qRT-PCR (primers are listed in Additional file 11: Table S6) on a QuantStudio 5 real-time PCR instrument (ThermoFisher, USA) with 2X SYBR Green Pro Taq HS Premix II (AGbio, China) was used to quantify the cDNA products. The mRNA expression was normalized to glyceraldehyde 3-phosphate dehydrogenase, while the miRNA expression was normalized to U6 small nuclear RNA. The relative expression between different samples was determined by the 2^−ΔΔCt^ method.

**RNase R and actinomycin D assays**

RNase and actinomycin D assays were used to determine the RNA stability. For the RNase R assay, 2 μg RNA was treated with 3 U/μg RNase R (BioVision, China) or diethylpyrocarbonate (DEPC)-treated water (control) at 37°C for 30 min. Thereafter, RNA was extracted with an RNeasy MinElute Cleaning Kit (Qiagen, Germany) and subjected to qRT-PCR quantification. For the actinomycin D assay, cells were treated with 2 μg/ml actinomycin D (Aladdin, China) to halt RNA synthesis. The remaining RNA in the cells was extracted and subjected to qRT-PCR quantification.

**DNA electrophoresis**

Genomic DNA (gDNA) was extracted using a kit (Sangon Biotech, China) according to manufacturer’s protocol. DNA electrophoresis of the PCR products was performed on a 2% agarose gels and stained using Safe Green (Biosharp).

**Immunoblotting**

Cells were lysed in ice-cold radioimmunoprecipitation assay (RIPA) buffer (ThermoFisher) with proteinase inhibitor (Beyotime). The lysate was centrifuged and the supernatant was collected. The protein concentration was determined by bicinchoninic acid assay (ThermoFisher). After performing sodium dodecyl sulfate polyacrylamide gel electrophoresis (SDS-PAGE), the proteins were transferred from the gel to a polyvinylidene fluoride membrane (Roche, USA), blocked with non-fat milk, and washed with phosphate-buffered saline (PBS). Specific primary antibodies were incubated with the membrane at 4 °C overnight. After washing with PBS, secondary anti-mouse/rabbit IgG were incubated with the membrane. Immunoblotting substrate (ThermoFisher) was added and the signal was detected using a FluorChem E System (General Electric, USA). Antibodies used in immunoblotting are as followed: HOXA5 (sc-365784, Santa Cruz, USA), Flag (F7425, Sigma-Aldrich, USA), N-cadherin (22018-1-AP, Proteintech, China), E-cadherin (20874-1-AP, Proteintech), Vimentin (5741, Cell Signaling Technology, USA), Twist (69366, Cell Signaling Technology), Snail (3879, Cell Signaling Technology), Slug (9585, Cell Signaling Technology), Zeb1 (21544-1-AP, Proteintech), β-catenin (51067-2-AP, Proteintech), DNMT3A (20954-1-AP, Proteintech), DNMT3B (26971-1-AP, Proteintech), DNMT3L (14939-1-AP, Proteintech), DNMT1 (ab188453, Abcam, USA), GAPDH (5174, Cell Signaling Technology), HRP-conjugated goat anti-mouse (SA00001-1, Proteintech), and HRP-conjugated goat anti-rabbit antibody (SA00001-2, Proteintech).

**Hematoxylin and eosin (HE) and immunohistochemical (IHC) staining**

For HE and IHC staining, 5-μm sections were incubated at 60°C for 2 h, soaked in xylene for deparaffinization, and washed with decreasing concentrations of alcohol for rehydration. For HE staining, the sections were incubated with hematoxylin and then eosin (Solarbio, China), followed by increasing concentrations of alcohol for dehydration. For IHC staining, the sections were microwaved in citrate solution for antigen retrieval (Solarbio), followed by adding 3% hydrogen peroxide (Solarbio) to inactivate endogenous peroxidase. After blocking with 5% bovine serum albumin (Beyotime) for 1 h, primary antibody were added and incubated overnight at 4°C. The unbound primary antibodies were washed off before adding the secondary antibodies. The signal was detected with a 3,3'-diaminobenzidine (DAB) substrate kit (Zhongshan Jinqiao, China) and the nuclei were stained with hematoxylin (Solarbio). The slides were independently evaluated by two pathologists. The scoring system was as follows: 1 point for 1–25% staining, 2 points for 26–50% staining, 3 points for 51–75% staining, and 4 points for 76–100% staining. Antibodies used in IHC are as followed: HOXA5 (sc-365784, Santa Cruz).

**Transwell migration and invasion assays**

To assess the *in vitro* cell migration and invasion ability, first, the cells were switched to serum-free medium for 12 h to achieve starvation and then re-suspended in serum-free medium to reach 1×10^6^ cells/ml. For the migration assay, 100 μl cells were added to transwell inserts (Corning, USA) and placed in a 24-well plate (Corning) with medium supplemented with 10% FBS in the lower chamber. For the invasion assay, the inserts were coated with 2% Matrigel (Corning) before adding the cells. After incubation, the inserts were placed in 4% polyformaldehyde (Biosharp) for fixation, washed with PBS, and stained with 0.4% crystal violet (Beyotime). Cells on the upper surface were discarded using a cotton swab. The migrated or invaded cells on the lower surface were counted based on five random high-power fields with an IX83 inverted microscope (Olympus, Japan).

**Cell Counting Kit-8 (CCK-8) assay**

CCK-8 assay was performed to evaluate the proliferative ability of cells. Briefly, 1×10^3^ cells in different treatment conditions were seeded into each well of a 96-well plate. 10 μl CCK-8 reagent (Biosharp) was added to each well and incubated for 1 h. After brief shaking of the plate, the absorbance at 450 nm was assessed using a Varioskan LUX machine (ThermoFisher).

**Immunofluorescence (IF) assay**

Prior to performing IF assays, 293 cells were seeded onto 15-mm glass-bottom plates (Nest, China). After the cells became attached, each plate was washed with PBS three times. The cells were fixed with 4% polyformaldehyde (Biosharp) for 30 min, permeabilized with 0.5% Triton X-100/PBS for 15 min, blocked with 5% bovine serum albumin (Beyotime) for 1 h, and then incubated with primary antibodies overnight at 4°C. After thoroughly washing off the unbound antibodies with PBS, the cells were incubated with secondary antibodies conjugated with fluorescent molecules at room temperature for 1 h. The nuclei were stained with 4',6-diamidino-2-phenylindole (DAPI) for 5 min. Fluorescence images were captured on an IX83 inverted microscope (Olympus). Antibodies used in IF are as followed: HOXA5 (sc-365784, Santa Cruz), DNMT3A (20954-1-AP, Proteintech).

**Fluorescence in situ hybridization (FISH) assay**

For FISH assays, probe hybridization was conducted using a special kit (GenePharma, China). Briefly, cells on a glass-bottom plate (Nest) were fixed with 4% polyformaldehyde (Biosharp) for 30 min, followed by washing with PBS. The probes were denatured at 75°C and mixed with hybridization buffer. Next, the probe mixture was added to the cells and incubated overnight at 37°C. After washing with PBS and DAPI staining, the cells were examined under an FV1000 confocal laser scanning microscope (Olympus).
