# Supplementary figures and images for: Correction: CircNTNG1 inhibits renal cell carcinoma progression via HOXA5-mediated epigenetic silencing of Slug
Source: Mol Cancer. 2023 Jan 20;22:14. doi: 10.1186/s12943-023-01726-w (PMC9854095; doi:10.1186/s12943-023-01726-w)

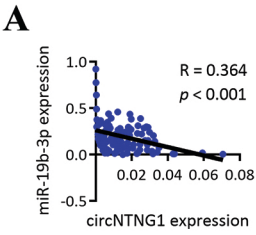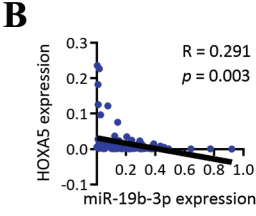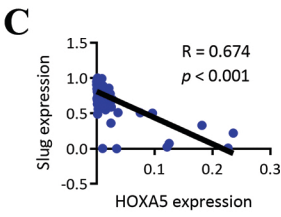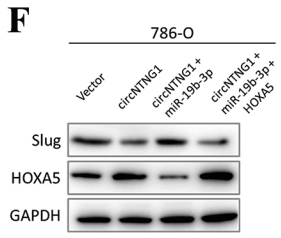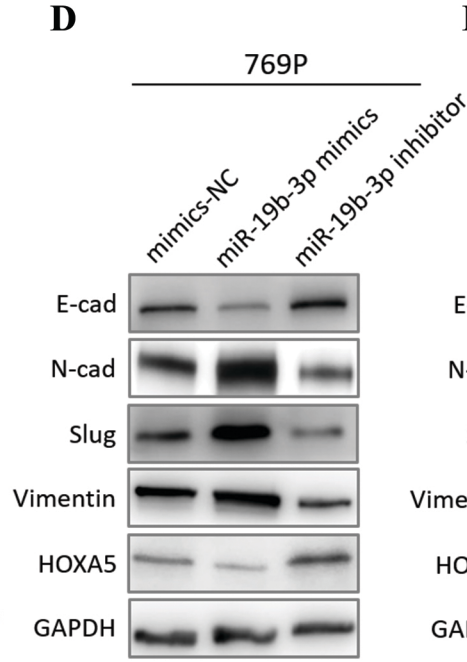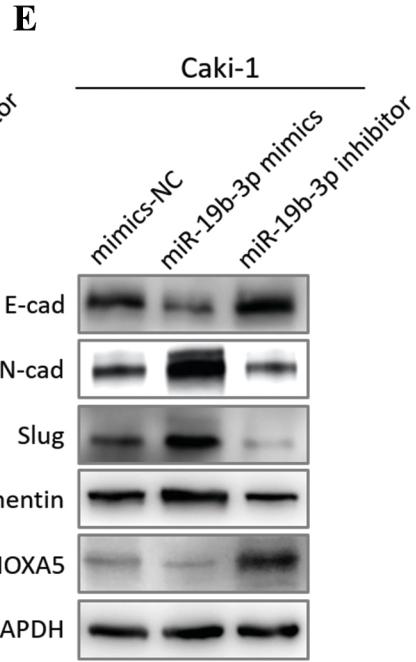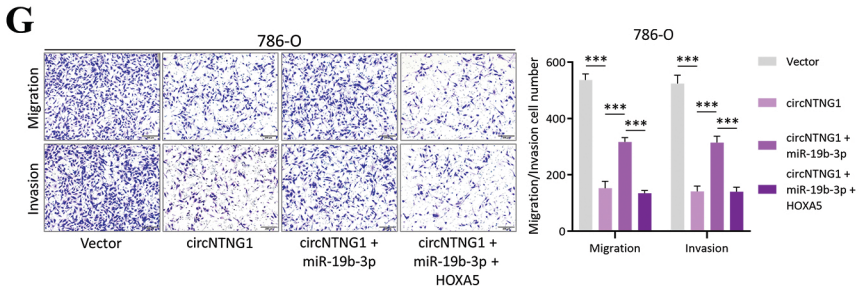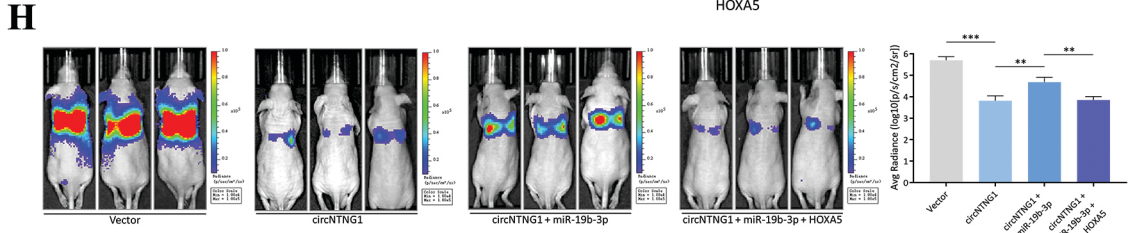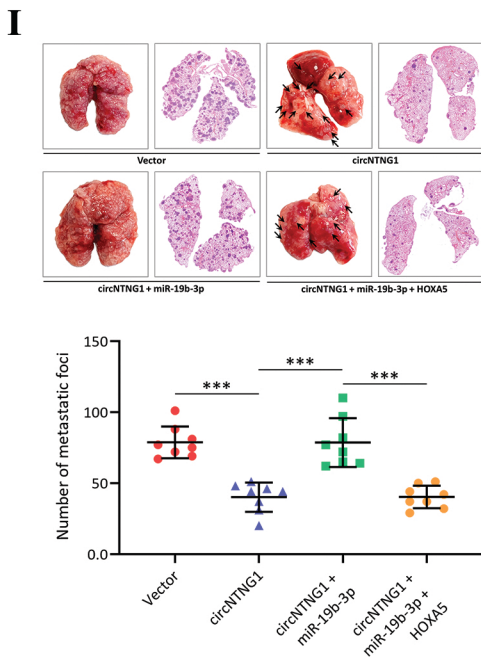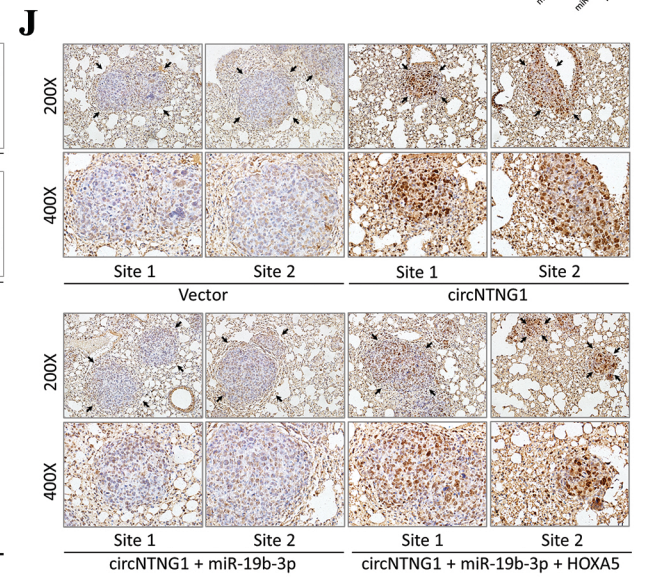

Supplement: Supplementary file 1 — Additional file 5: Fig. S5. The regulation of RCC by circNTNG1/miR-19b-3p/HOXA5 axis. A. Correlation analysis of circNTNG1 and miR-19b-3p in our own patient cohort (102 patients). B. Correlation analysis of miR-19b-3p and HOXA5 in our own patient cohort (102 patients). C. Correlation analysis of HOXA5 and Slug in our own patient cohort (102 patients). D. Immunoblotting of HOXA5, Slug and EMT markers with treatment of miR-19b-3p mimics or miR-19b-3p inhibitor in 769P cells. GAPDH was used as internal control. E. Immunoblotting of HOXA5, Slug and EMT markers with treatment of miR-19b-3p mimics or miR-19b-3p inhibitor in Caki-1 cells. GAPDH was used as positive control. F. Immunoblotting of HOXA5 and Slug in 786-O cells circNTNG1 overexpression salvage experiment. Cells were treated with circNTNG1 overexpression with/without miR-19b-3p mimics, and further salvaged with HOXA5 overexpression. GAPDH was used as positive control. G. Representative images (left) and quantification (right) data of Transwell migration/invasion assay of 786-O cells circNTNG1 overexpression salvage experiment. H. Representative images (left) and in vivo luciferase activity quantification (right) data of mouse tail-vein lung metastasis model. 786-O cells with vector, circNTNG1-overexpression, circNTNG1-overexpression+miR-19b-3p overexpression or circNTNG1-overexpression+miR-19b-3p overexpression+HOXA5 overexpression were used in the injection. Images were taken 8 weeks after the injection. I. Representative HE-stain images (left) and lung-metastasis foci quantification (right) data of 786-O mouse tail-vein lung metastasis model. J. Representative IHC images of HOXA5 in lung-metastasis foci from 786-O mouse tail-vein lung metastasis model. Data are mean ± SD, n = 3. [file 12943_2023_1726_MOESM1_ESM.pdf]
